# Quantitative analysis of *P**lasmodium* ookinete motion in three dimensions suggests a critical role for cell shape in the biomechanics of malaria parasite gliding motility

**DOI:** 10.1111/cmi.12283

**Published:** 2014-03-28

**Authors:** Andrey Kan, Yan‐Hong Tan, Fiona Angrisano, Eric Hanssen, Kelly L. Rogers, Lachlan Whitehead, Vanessa P. Mollard, Anton Cozijnsen, Michael J. Delves, Simon Crawford, Robert E. Sinden, Geoffrey I. McFadden, Christopher Leckie, James Bailey, Jake Baum

**Affiliations:** ^1^ Victoria Research Laboratory, National ICT Australia (NICTA)Department of Computing and Information SystemsUniversity of MelbourneMelbourneVic.3010Australia; ^2^ Infection and Immunity DivisionThe Walter and Eliza Hall of Institute of Medical ResearchParkvilleVic.3052Australia; ^3^ Centre for Dynamic ImagingThe Walter and Eliza Hall of Institute of Medical ResearchParkvilleVic.3052Australia; ^4^ Department of Medical BiologyUniversity of MelbourneVic.3052Australia; ^5^ Electron Microscopy Unit Bio21 Molecular Science and Biotechnology Institute and Department of Biochemistry and Molecular BiologyUniversity of MelbourneParkvilleVic.3010Australia; ^6^ School of BotanyThe University of MelbourneParkvilleVic.3010Australia; ^7^ Department of Life SciencesImperial College of Science, Technology and MedicineLondonSW7 2AZUK; ^8^ Department of Life SciencesSir Alexander Fleming BuildingImperial College LondonSouth KensingtonLondonSW7 2AZUK

## Abstract

Motility is a fundamental part of cellular life and survival, including for *P**lasmodium* parasites – single‐celled protozoan pathogens responsible for human malaria. The motile life cycle forms achieve motility, called gliding, via the activity of an internal actomyosin motor. Although gliding is based on the well‐studied system of actin and myosin, its core biomechanics are not completely understood. Currently accepted models suggest it results from a specifically organized cellular motor that produces a rearward directional force. When linked to surface‐bound adhesins, this force is passaged to the cell posterior, propelling the parasite forwards. Gliding motility is observed in all three life cycle stages of *P**lasmodium*: sporozoites, merozoites and ookinetes. However, it is only the ookinetes – formed inside the midgut of infected mosquitoes – that display continuous gliding without the necessity of host cell entry. This makes them ideal candidates for invasion‐free biomechanical analysis. Here we apply a plate‐based imaging approach to study ookinete motion in three‐dimensional (3D) space to understand *P**lasmodium* cell motility and how movement facilitates midgut colonization. Using single‐cell tracking and numerical analysis of parasite motion in 3D, our analysis demonstrates that ookinetes move with a conserved left‐handed helical trajectory. Investigation of cell morphology suggests this trajectory may be based on the ookinete subpellicular cytoskeleton, with complementary whole and subcellular electron microscopy showing that, like their motion paths, ookinetes share a conserved left‐handed corkscrew shape and underlying twisted microtubular architecture. Through comparisons of 3D movement between wild‐type ookinetes and a cytoskeleton‐knockout mutant we demonstrate that perturbation of cell shape changes motion from helical to broadly linear. Therefore, while the precise linkages between cellular architecture and actomyosin motor organization remain unknown, our analysis suggests that the molecular basis of cell shape may, in addition to motor force, be a key adaptive strategy for malaria parasite dissemination and, as such, transmission.

## Introduction

Malaria is one of the leading causes of infant mortality in the developing world (WHO, 2013). The disease is caused by intracellular parasites from the genus *Plasmodium*, single‐celled protozoa that are transmitted to a vertebrate host following the bite from an infected female mosquito. Its two‐host life cycle – progressing from the midgut of an infected mosquito, through to a vertebrate host and back into the subsequent feeding mosquito – is dominated by cell motility. At every stage, the parasites must target and penetrate host tissues to establish the sequential steps of development (Prudencio *et al*., 2006; Vlachou *et al*., 2006; Cowman *et al*., 2012). Unlike other eukaryotic cells, however, cell motility in these parasites does not involve gross changes in cell morphology or the use of a flagellum [though they are certainly capable of generating one as is shown with male gametocytes (Sinden and Croll, 1975)], but is instead driven by an internal actomyosin motor, which allows the motile parasite to literally glide across substrate surfaces and invade host cells (Soldati *et al*., 2004; Baum *et al*., 2008; Farrow *et al*., 2011). The molecular basis for gliding motility in *Plasmodium* has been studied most intensively in the two life cycle stages associated with intracellular infection of the vertebrate host (host‐cell invasion), the sporozoite and merozoite (Kappe *et al*., 1999; Baum *et al*., 2006). Work on these stages, drawing extensively on studies from the related apicomplexan parasite *Toxoplasma gondii* (Gaskins *et al*., 2004), has identified several core components of the gliding motor in addition to actin and myosin. This gliding‐associated complex (or glideosome) anchors the myosin motor to the external membrane of a flattened leaflet that lies within the parasite pellicle, called the inner membrane complex (IMC) (Raibaud *et al*., 2001). The proteins that make up the glideosome are broadly conserved across the apicomplexan phylum to which *Plasmodium* parasites belong (Bergman *et al*., 2003; Gaskins *et al*., 2004; Baum *et al*., 2006; Green *et al*., 2006; Jones *et al*., 2006; Frénal *et al*., 2010). According to current thinking, these link through to the extracellular milieu via a series of linkages beginning with the short actin filament (Russell and Sinden, 1981; King, 1988), the glycolytic enzyme aldolase (Buscaglia *et al*., 2003; Jewett and Sibley, 2003), and finally surface‐secreted transmembrane‐associated adhesins (Buscaglia *et al*., 2003; Jewett and Sibley, 2003; Baum *et al*., 2006; Starnes *et al*., 2009). The combined rearward passage of linked adhesins, by the myosin motor, propels the parasite along substrate surfaces and/or into host cells (Pinder *et al*., 1998; Meissner *et al*., 2002; Siden‐Kiamos *et al*., 2011).

Despite its extensive study, there is only limited evidence for the physical organization of the motor and glideosome components within the parasite cell other than their general spatial compartmentalization, or their specific membrane association (Frénal *et al*., 2010). A suggestion for organization in the IMC may be visible in cryoelectron microscopy (CryoEM) of the *Plasmodium gallinaceum* ookinete (Raibaud *et al*., 2001). Imaging of this life cycle stage, the motile polyploid zygote that colonizes the mosquito midgut (Angrisano *et al*., 2012c), reveals a periodic distribution of inner membrane particles (Raibaud *et al*., 2001) that would fit with an organized distribution of the motor in the pellicular space. Evidence for actual organization of each motor component has, however, relied primarily on immunoprecipitation of glideosome and associated constituents, stepwise links between which bridge the gap from the IMC inside through to the extracellular milieu (Kappe *et al*., 1999; Bergman *et al*., 2003; Gaskins *et al*., 2004; Baum *et al*., 2006; Green *et al*., 2006; Jones *et al*., 2006; Frénal *et al*., 2010). Critically, because of lack of evidence of organization, it is unclear how precise directional force is generated, for example whether it is via the orientation of actin filaments (F‐actin), the myosin motor head or some other guiding constraint (Raibaud *et al*., 2001). A key limitation is that no study has been able to visualize F‐actin in the pellicular space of a motile apicomplexan parasite, whose orientation would logically be a key determinant of effective cell movement (Wetzel *et al*., 2003; Kudryashev *et al*., 2010; Angrisano *et al*., 2012b). Reasons for this may be due to the short and highly dynamic nature of *Plasmodium* actin microfilaments (Schmitz *et al*., 2005; Schüler *et al*., 2005; Sahoo *et al*., 2006) coupled with the relatively small size of motile parasite cells that contain them, rendering current visualization techniques less feasible to utilize (Schmitz *et al*., 2005; Schüler *et al*., 2005; Sahoo *et al*., 2006). Myosin complex organization has not been addressed at sufficient resolution to assess its contribution. Thus, our understanding of how molecular changes inside the parasite translate into directional force remains speculative.

The fundamentals of gliding have instead been largely derived at whole cell level, exploring motile parasites *in vitro* and *in vivo* by video microscopy (King, 1981; 1988; Russell and Sinden, 1981; Wetzel *et al*., 2003). Cell shape in particular would be expected to play a significant role in the biomechanics of gliding, as found with other unicellular pathogens such as trypanosomatids and spirochaetes (Murphy *et al*., 2008; Heddergott *et al*., 2012). Towards this, studies on the ultrastructure of sporozoites have found that cellular asymmetry likely underlies chiral motion of this parasite stage (Kudryashev *et al*., 2012). Chiral shape does not appear to hold true for the merozoite beyond an asymmetrical distribution of microtubules (Hanssen *et al*., 2013) and the question of three‐dimensional (3D) shape in the ookinete has not been fully explored.

Video microscopy has been instrumental in defining the key role of actin in *Plasmodium* and related parasite cell movement, such as via bead movement on the surface of parasites or their general arrest in the presence of actin inhibitors (King, 1988; Siden‐Kiamos *et al*., 2006; Munter *et al*., 2009; Angrisano *et al*., 2012a). Most recently, detailed imaging of *Plasmodium berghei* sporozoites has revealed that cell adhesion to the substrate and a continuous sequence of actin‐dependent stick‐and‐slip phases of motility underlies sporozoite actomyosin‐based motility (Munter *et al*., 2009; Hegge *et al*., 2010) making a linkage between parasite molecular events and its effect on motion. Likewise, studies with a hydrogel system have made steps towards defining the first *in vivo* estimates of motor organization, such as actin filament length, by varying inter‐particle spacing in these artificial grids (Perschmann *et al*., 2011). Despite these detailed studies, however, resolving the long‐standing question of the interplay between motor organization, cell shape and directional motility has not been achieved and, as importantly, only a few studies have attempted to explore cell motility in a 3D context for any apicomplexan cell (Vlachou *et al*., 2004; Amino *et al*., 2006; Hellmann *et al*., 2011).

The ookinete while known to rely on the same basic actomyosin motor for movement (Dessens *et al*., 1999; Yuda *et al*., 1999; Siden‐Kiamos *et al*., 2006; 2011; Angrisano *et al*., 2012a), has been much less investigated compared to its blood and liver‐stage counterparts. Unlike sporozoite and merozoite stages, the ookinete relies on continuous motile forces to break through the epithelial cell layer of the midgut, during which the ‘invaded’ cell is lysed (Han *et al*., 2000; Vlachou *et al*., 2004). It may thus serve as an ideal representative cell for dissecting the mechanics of general motility in *Plasmodium* cells (and Apicomplexa more broadly) given the absence of potentially confounding influences of the host cell during invasion. Ookinetes form in the blood bolus 10 to 25 h post gamete fusion (syngamy) (Janse *et al*., 1985). They must reach the mosquito midgut epithelium and burrow through to the basal lamina, with very few completing development into an oocyst (Baton and Ranford‐Cartwright, 2005; Sinden, 2009). Although some of the molecular events involved in midgut traversal have been defined (Kariu *et al*., 2006; Armistead *et al*., 2011; Mathias *et al*., 2012), the process of ookinete targeting of the epithelium is still incompletely understood. The distribution of gametocytes, forming zygotes and resulting ookinetes, would be expected to be approximately random within the blood bolus; however, targeting of the midgut could be either stochastic or involve a chemotactic or cellular trophic signal. While some studies have suggested the possibility of ookinete tropism for certain cells within the midgut epithelium (Shahabuddin *et al*., 1998; Cociancich *et al*., 1999), this hypothesis, or indeed any obvious chemotactic behaviour from the ookinete, is not supported from *in vivo* or *ex vivo* video microscopy of the colonization process (Freyvogel, 1966; Zieler and Dvorak, 2000; Vlachou *et al*., 2004). Thus, in the absence of cellular tropism, and given that digestion of the blood meal and ookinete colonization of the midgut epithelium basal layer largely coincide [at around 24 h post feeding (Graf *et al*., 1986)], we might expect exit from the central blood meal and oocyst formation to occur largely at random along the epithelial surface.

Ookinete motility itself *in vivo, ex vivo* (Freyvogel, 1966; Zieler and Dvorak, 2000; Vlachou *et al*., 2004) and *in vitro* (Moon *et al*., 2009; Angrisano *et al*., 2012a; Volkmann *et al*., 2012) has been characterized as ranging from a general ‘snake‐like’ helical pattern defining the most obvious motile event to other spiralling or linear forms of motion, the significance of which is not clear. However, no study to date has described a quantitative model for ookinete motility or defined how movement in 3D might correlate to traction and force from the internal actin‐myosin motor. In addition, while the role of cell shape in ookinete motility is clearly important (Tremp and Dessens, 2011; Volkmann *et al*., 2012), links between the three key properties of motility, cell shape and directional motion have not been made.

Here, we combine insights about the spatial distribution of ookinete colonization of the mosquito midgut, a plate‐based assay for *in vitro* motility and 3D structural microscopy to advance the ookinete as a powerful life cycle cell type to test the fundamental principles of *Plasmodium* cell motility and assess contributions of cell shape and motor force in determining the biomechanics of parasite movement that underpin successful transmission.

## Results and discussion

### Spatial distribution of ookinete exit from the midgut

**Figure 1:**
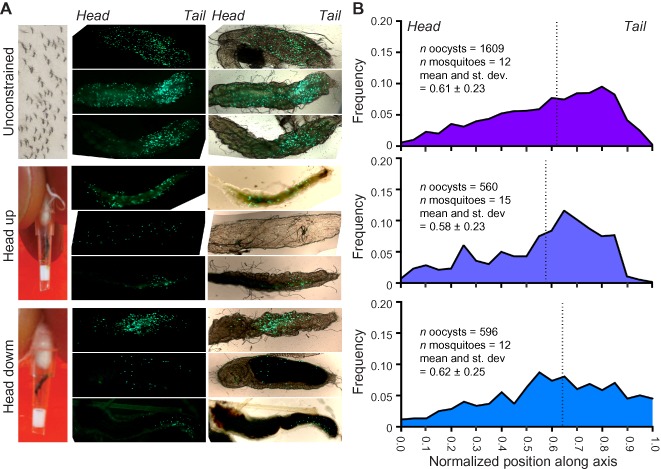
Colonization of the mosquito midgut by *P**. berghei* ookinetes is effectively random. A. Post‐feed mosquitoes were allowed to rest unconstrained or immobilized in head‐down or head‐up orientations. The distribution of fluorescent oocysts was measured along the anterior–posterior axis 5 days post feeding. B. Frequency distributions of individual oocysts along anterior–posterior axis is similar across the three groups of mosquitoes irrespective of immobilized orientation.

It remains uncertain whether ookinetes in native infections target a subset of midgut cells (showing specific host‐cell or tissue tropism) or exit the midgut stochastically as a result of random formation and exit from the blood meal (Sinden and Billingsley, 2001). One reason for confusion in this area is that the distribution of ookinete exit may be confounded by gravity, whereby oocysts preferentially develop at the posterior end of the midgut due to either the vertical (head‐up) post‐feed resting of engorged mosquitoes (Cociancich *et al*., 1999; Han *et al*., 2000; Sinden and Billingsley, 2001), the progression of digestion from posterior to anterior in the midgut (Graf *et al*., 1986) or any difference in density that they show when compared to the surrounding blood meal. Lack of clarity about this alters the expectation of whether ookinetes are expected to demonstrate classical foraging behaviour, such as random walks, tumbling or true chemotaxis to facilitate midgut exit (Berg, 1976), or follow a relatively simple (and energy efficient) strategy of moving along a straight line until they reach the epithelium. To explore which strategy may be in action, we determined the spatial distribution of *P. berghei* oocysts in multiple infected *Anopheles stephensi* mosquitoes 5 days post feeding. Although there will be some disconnect between ookinete crossing the midgut and oocyst formation – since ookinetes explore the midgut surface before crossing (Zieler and Dvorak, 2000; Vlachou *et al*., 2004) – oocyst distribution represents a good approximation for the spatial distribution of exit events. Previous studies have explored the question of cellular tropism in the midgut, by measuring oocyst distribution along the anterior–posterior axis in unconstrained mosquitoes versus those kept in a rotating chamber (Cociancich *et al*., 1999). We revisited these experiments by immobilizing mosquitoes in pipette tips in a restricted head‐up, head‐down or unconstrained (unbiased) position (Fig. 1A) using a *P. berghei* line constitutively expressing green fluorescent protein (GFP) (Franke‐Fayard *et al*., 2004). Five days post feeding, the earliest time at which fluorescent oocysts could be adequately resolved and latest to which mosquitoes tolerated immobilization, GFP foci were counted. A frequency distribution of these oocysts along the anterior–posterior axis showed no statistically significant bias between the test and unconstrained feeding regimens (mean and SD: unconstrained = 0.61 ± 0.23; head up = 0.58 ± 0.23, head down = 0.62 ± 0.25, Mann–Whitney *U*‐test between means, unconstrained versus head up or head down groups, *P* = 0.87 and 0.93, respectively, Fig. 1B). The broad spatial distribution, independent of immobilized‐mosquito orientation, therefore suggests that ookinete exit from the midgut is not influenced by gravity or density. Furthermore, while a trend of the distribution towards the posterior midgut is consistent with most parasites developing within the bulk of the blood meal (that sits posteriorly), no other obvious tissue tropism or targeting is evident, consistent with the work of others (Han *et al*., 2000; Zieler and Dvorak, 2000). We therefore believe it is most likely that ookinetes exit the midgut in an unbiased or random fashion, dependent only on their site of formation within the blood meal itself.

### An *in vitro*, quantitative 3D ookinete motility assay

If exit is unbiased, for randomly oriented ookinetes that are unaware of their position within the midgut, a simple straight line exiting strategy would appear to be the most efficient way of reaching the midgut epithelium. For example, for a continuous time random walk (Metzler and Klafter, 2000) there is a probability that exiting would take a considerable amount of time, whereas straight line motion guarantees to find an exit within ∼ 2 h (given an average cross‐section width or length of the midgut between ∼ 0.8 mm and 1.2 mm and apparent speed of ∼ 10 μm min^−1^, see next section). Previous video microscopy of motility, however, clearly demonstrates that ookinetes move with a complex non‐linear motion trajectory. Ookinetes from several species have been found to move in a meandering helical fashion both *in vitro* and *in vivo* suggesting other factors influence epithelial targeting beyond a simple straight‐line exit strategy (Freyvogel, 1966; Vlachou *et al*., 2004; Moon *et al*., 2009; Ramakrishnan *et al*., 2011; Volkmann *et al*., 2012). A single study described three distinct patterns of motility: stationary rotation, directional spiralling and straight‐segment motility (Vlachou *et al*., 2004). However, there is still insufficient detail in these data to explore whether a general model for motility is applicable to ookinetes that could be used towards defining some of its underlying mechanisms.

**Figure 2:**
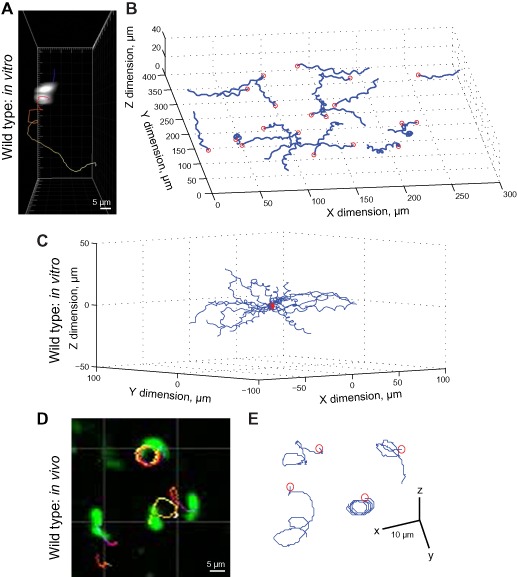
Parameterization of *P**. berghei* ookinete cell movement by *in vitro* and *in vivo* three dimensional motility assays. A and B. (A) A sample 3D ookinete track in Matrigel and (B) reconstructed 3D tracks from a single well Matrigel assay exhibiting characteristic helical motion paths. C. Reconstructed tracks *in vitro* from panel (B) translocated to a common origin do not show any prominent bias in direction. D and E. Imaging and single motion tracks for ookinetes imaged in an explanted *Anopheles stephensi* midgut. Red circles in tracks denote the initial position of fluorescent ookinetes (see also Supplementary Movies S1–S4).

To explore whether ookinete movement can be described in general terms, we extended the current *in vitro* Matrigel assay (Ishino *et al*., 2006; Moon *et al*., 2009; Volkmann *et al*., 2012) towards a more quantitative format that could measure ookinete motion in 3D space and use the parameters of this movement to build a model for idealized ookinete motility (see *Experimental procedures*). Reconstruction of tracks in 3D clearly confirmed the characteristic helical motion paths for ookinetes (Fig. 2A and B and Supplementary Movies S1–S3). Re‐centring motility tracks in each assay at a single point, demonstrated that motion is random in 3D space, showing no overall bias in directionality (Fig. 2C; Wilcoxon signed‐rank test for the differences between x, y or z co‐ordinates for each track at the beginning and end of movie *P*‐values 0.30, 0.72 and 0.09, respectively, *n* = 52; see Supplementary Discussion for extended discussion). While obviously lacking potential host chemotactic cues in our assay format, the random distribution is consistent with our imaging of the entire infected midgut excluding gravitational forces in the colonization process. Finally, although predominantly focused on an *in vitro* description of motility, we wanted to explore whether motion in a Matrigel assay is qualitatively similar to *in vivo* ookinete motility. Towards assessing this, 3D tracks were reconstructed from real‐time *in vivo* imaging of the same fluorescent ookinetes travelling through an explanted infected *A. stephensi* mosquito midgut. Because of difficulty in controlling the timing of ookinete development and mosquito infections, only a few events were captured that could facilitate detailed description (*n* = 4). However, of those captured, chiral motion was clearly apparent (Fig. 2D and E and Supplementary Movies S4 and S5) and, while speed was reduced (e.g. apparent speed ∼ 10.5 *in vitro* versus ∼ 7 μm min^−1^ *in vivo*), the approximate coherence between our *in vitro* and *in vivo* imaging data is consistent with motion paths seen with previous *in vitro* and *in vivo* work (Freyvogel, 1966; Vlachou *et al*., 2004; Volkmann *et al*., 2012).

In summary, following the absence of evidence for a preferred site of exit within the mosquito midgut (previous section) our *in vitro* motility assay did not reveal any apparent bias in directionality, nor the change in directionality over time, which combined are concordant with a ‘straight line’ exit strategy used by the ookinete.

### Parameterization of ookinete motility

While variation in motility is clearly present, the mode of movement found in the majority of moving ookinetes could be adequately described by six parameters (see Supplementary Discussion, Supplementary Figs S1–S3 and Supplementary Code and Data, available on request): apparent speed (μm min^−1^), total displacement (μm), chirality (% of left‐handed turns), period of the helix (min, time taken to make a loop), radius of the helix (μm) and pitch of the helix (μm) (Table 1).

**Table 1:** Numerical description of ookinete locomotion using six parameters

	Apparent speed	Total displacement (10 min)	Chirality	Period	Radius	Pitch
	(μm min^−1^)	(μm)	(% left turns)	(min)	(μm)	(μm)
Wild‐type	10.5 ± 2.3	66.3 ± 16.6	80.0 ± 20.8	1.6 ± 0.4	2.2 ± 0.5	12.6 ± 4.2
	Total tracks = 52			Helical tracks = 43		
100 nM CD	4.3 ± 1.15	17.4 ± 11.2	11.8 ± 20.8	2.4 ± 0.1	2.1 ± 0.4	8.0 ± 1.5
	Total tracks = 56			Helical tracks = 6		
IMC1h‐KO	7.5 ± 3.1	28.2 ± 16.4	18.8 ± 11.2	–	–	–
	Total tracks = 39			Helical tracks = 0		
Wild‐type *in vivo*	7.0 ± 1.0	10.7 ± 6.2	78.6 ± 26.4	3.7 ± 1.1	2.9 ± 0.7	4.4 ± 2.9
	Total tracks = 4			Helical tracks = 4		

If period estimation fails a track is considered erratic, and period is not reported. Furthermore, radius and pitch are not computed for erratic tracks. Numbers show mean ± SD.

Apparent speed is an estimation of the real (instantaneous) ookinete speed, calculated as a sum of distances between track locations in subsequent images divided by the total travel time (as opposed to the gross distance travelled by an ookinete over time, see Supplementary Discussion). In our assay, ookinetes moved with an estimated speed of 10.5 ± 2.3 μm min^−1^ (mean and SD, Table 1). This figure is faster than previous estimates for ookinete speed in Matrigel [5.8 μm min^−1^ (Moon *et al*., 2009), 6.8 μm min^−1^ (Volkmann *et al*., 2012)], insect cell co‐culture [6 μm min^−1^ (Siden‐Kiamos *et al*., 2006)] and *in vivo* midgut assays [5 μm min^−1^ (Vlachou *et al*., 2004) and 7 μm min^−1^ from our own *in vivo* imaging, Table 1]. Of note, these estimates (except our own *in vivo* analyses) were obtained using notably lower frame rates. Furthermore, some of these estimates reflect total displacement divided by time. Both factors will combine to underestimate total distance covered and thus reduce the actual speed determined (Supplementary Discussion). For example, in our assay total net displacement over 10 min was found to be 66.3 ± 16.6 μm (Table 1), implying an average speed of 6.6 μm min^−1^, directly comparable with previous measures. However, given our own slower speeds for *in vivo* imaging (Table 1) we cannot exclude the possibility that density of the extracellular environment may be inconsistent between our Matrigel assay and others described. Ultimately, while acknowledging the variance associated with our own measurements, a speed of approximately 10 μm min^−1^ is a good approximation of ookinete instantaneous speed in these artificial conditions.

Chirality measures how well tracks conform to a right‐handed helix (values close to −100%) or to a left‐handed helix (values close to +100%), where 0 would indicate an erratic track (i.e. no prominent helical pattern). Helical motion is left‐handed when the path of motion along the helix's axis moves towards the observer following a clockwise screwing motion. In our assay, chirality was 80.0 ± 20.8% (Table 1) demonstrating that ookinetes almost exclusively move in a *left*‐handed helix. Of note, studies with *T. gondii* have found a similar left‐handed chirality to their 3D helical motion (Leung *et al*., 2014) suggesting general apicomplexan principles may be at work. Previous studies on *Plasmodium* sporozoite motion, focused on two‐dimensional (2D) measurements of parasite motion on a glass slide, described motility in terms of clockwise or counter‐clockwise motion (Vanderberg, 1974; Russell and Sinden, 1981; Hegge *et al*., 2010; Kudryashev *et al*., 2012). However, from 2D data alone it is not possible to determine chirality of the underlying 3D helix (Gurarie *et al*., 2011).

**Figure 3:**
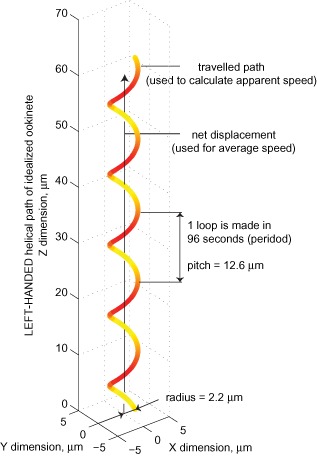
The left‐handed helical motion of *P**. berghei* ookinetes. An idealized model of left‐handed helical motion using the empirically defined parameters inferred from reconstructed tracks.

The last three parameters describe a helical motion model, where periodic motion is reliably established. Periodicity estimates the time it takes for an ookinete to make one complete loop, found to be on average 1.6 ± 0.4 min, while, radius (across the helical cylinder) and pitch (distance between loops) were found to be, 2.2 ± 0.5 μm and 12.6 ± 4.2 μm respectively (Table 1). These estimated statistics of helical motion can be presented as an idealized ookinete track describing the motion of the majority of motile parasite cells (Fig. 3). Where ookinetes move erratically or according to another unrelated gait (Vlachou *et al*., 2004), our periodicity estimation method cannot be applied (see *Experimental procedures*) and, logically, radius and pitch cannot be computed. Of note, radius and pitch estimates depend on computed period, but are independent of other parameters, while chirality, speed and displacement, are each computed independently. Chirality is internally based on the choice of step size (see Supplementary Discussion), but this does not affect any other parameter.

### Effects of actin inhibition on ookinete motility parameters

**Figure 4:**
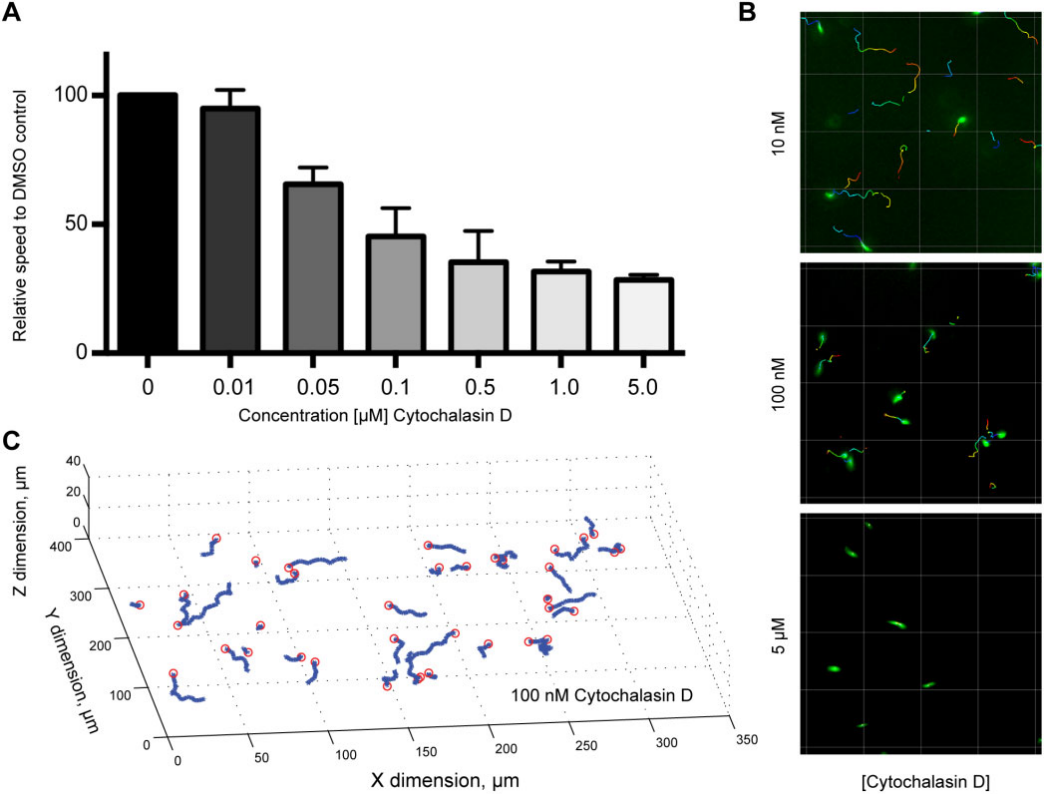
Cytochalasin D ablates *P**. berghei* ookinete motion. A. Dosage response curve of average ookinete speed (calculated from net displacement) in the presence of cytochalasin D, used to determine an effective concentration at which speed is reduced to 50% (∼ 100 nM). Data from three independent replicates, with mean and SD (error bars) shown. B. Representative 2D motion tracks for 10 nM, 100 nM (IC50) and 5 μM cytochalasin D treatment. Scale bar = 40 μm. C. Reconstructed 3D motion tracks from a representative motility assay well using 100 nM cytochalasin D.

Ookinete motion has been extensively described as being dependent on actin and myosin (Siden‐Kiamos *et al*., 2006; 2011; Angrisano *et al*., 2012a). To date, specific inhibitors of apicomplexan myosin have not been described. However, ookinete motility is sensitive to actin destabilizing drugs such as cytochalasin D (CD) (Siden‐Kiamos *et al*., 2006; 2011; Angrisano *et al*., 2012a). Ookinete average speed (estimated from net displacement) over a range of CD concentrations compared to dimethyl sulfoxide (DMSO) untreated controls was measured and found to lead to a ∼ 50% reduction in speed at 100 nM (Fig. 4A and B), in line with previous reports (Siden‐Kiamos *et al*., 2006; Angrisano *et al*., 2012a). At this concentration apparent speed was reduced from 10.5 ± 2.3 μm min^−1^ to 4.3 ± 1.2 μm min^−1^ compared to DMSO controls (Fig. 4C, Table 1). Total displacement under these conditions was markedly reduced compared to controls and chirality dropped down to 11.8 ± 20.8%. However, a reduction in chirality may primarily arise from a difficulty in assessing this parameter in immotile parasites. Of note, from the 56 tracks analysed, only six could be defined as predominantly chiral (Fig. 4, Table 1). These tracks, though short, were all left‐handed, had a very similar radius to those of untreated parasites (Mann–Whitney *U*‐test, *P* > 0.05) but had a lengthened period and reduced pitch (Mann–Whitney test, *P* < 0.001). Thus, while CD clearly ablates motility, it would seem that curtailing motor function does not change all gross parameters of ookinete motion.

### Assessing the cellular basis for ookinete chirality

Of all parameterized features of ookinete motion, the strict handedness of ookinetes in 3D space (80.0 ± 20.8%) is the most striking. This feature has been described in several previous reports of *Plasmodium* parasite cellular motility, although detail of image capture conditions and different nomenclatures used (clockwise instead of handedness for example) make it hard to correlate whether strict handedness of ookinetes (Speer *et al*., 1975; Vlachou *et al*., 2004) or sporozoites (Vanderberg, 1974; Kudryashev *et al*., 2012) is a conserved *Plasmodium* feature. Chirality as a cell motility phenomenon can arise from two (interdependent) cellular factors. Strict organization of the motor force that provides a consistently directed rotational force will give rise to generalized chiral, helical motion (Jennings, 1901; Jahn and Votta, 1972; Crenshaw, 1996). Alternatively, the shape of a motile cell will also give rise to chiral motion as it does with trypanosomes or spirochaetes (Murphy *et al*., 2008; Heddergott *et al*., 2012) irrespective of how the force is produced. In apicomplexan parasites, shape and motor organization are both inter‐linked by their dependence on the pellicular cytoskeleton, i.e. the subpellicular microtubules and subpellicular network (Mann and Beckers, 2001; Morrissette and Sibley, 2002), composed of alveolins (or IMC proteins) and other protein components (Raibaud *et al*., 2001; Gould *et al*., 2008; Bullen *et al*., 2009). Although the precise nature of linkages is not known, myosin motor and glideosome specifically connect with the subpellicular network (Bullen *et al*., 2009). At present, there is no clear way of disrupting glideosome organization without affecting its function to test whether it is the precise organization/anchorage of motor force, such as the myosin head or actin filament polarity, that drives directional parasite chiral motion or whether it is the general parasite shape or both.

**Figure 5:**
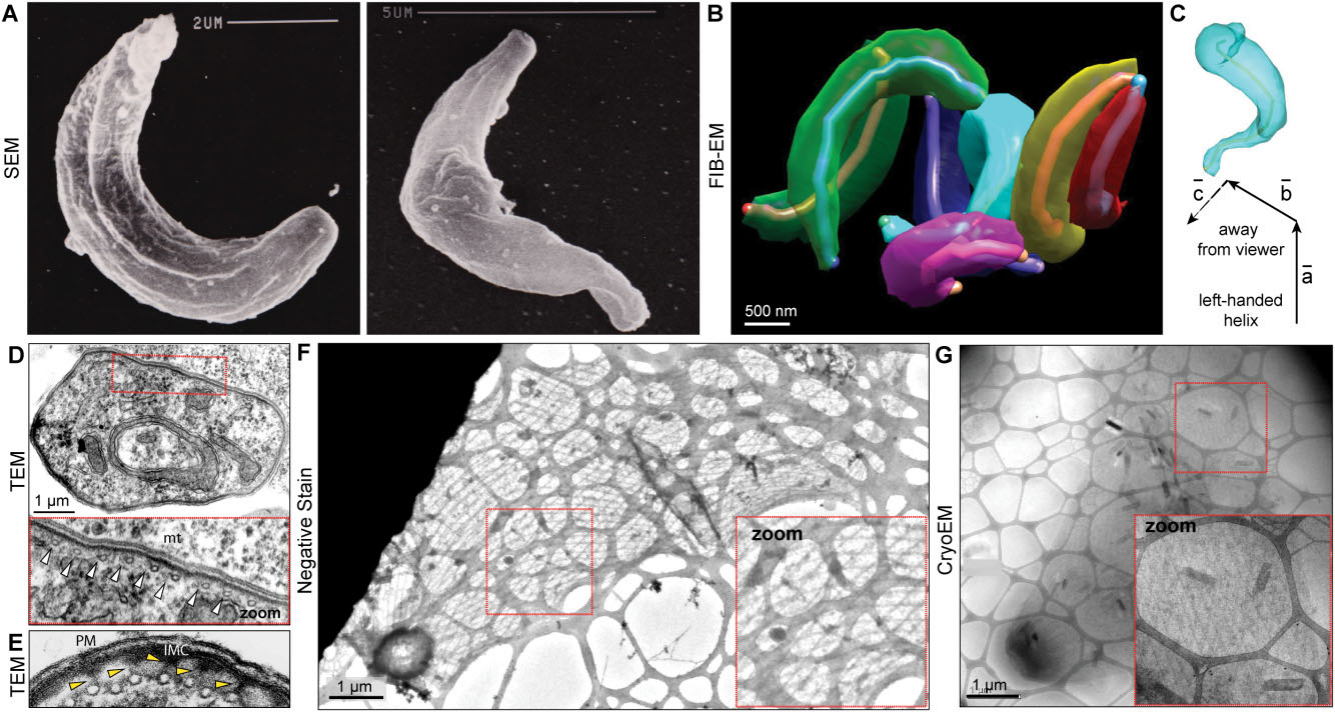
*P**. berghei* ookinetes have a distinctive 3D shape and underlying twisted microtubule lattice. A. Scanning electron microscopy (SEM) of ookinetes. B. 3D shape of ookinetes reconstructed using Focused Ion Beam‐Electron Microscopy (FIB‐EM) reveals chiral shape of ookinetes. Note, a pseudo‐skeletonized rod‐shaped line is traced along the ookinete central axis to help visualize curvature (i.e. twist or curl) of cell shape. C. A single ookinete from (B) conforming to a left‐handed helical element, where four consecutive points define three vectors (***a***, ***b***, ***c***) with a distinct spatial orientation (see Supplementary Discussion and Supplementary Fig. S2). D. Transmission electron microscopy (TEM) section showing circular array of subpellicular microtubules (zoom, white arrowheads point to microtubules, mt). E. As D showing electron dense linkages between subpellicular microtubules and inner membrane complex (IMC). PM: plasma membrane. F. Critical point‐dried cytoskeleton of a *P. berghei* ookinete visualized by negative staining electron microscopy reveals the underlying twisted microtubule lattice. Distinct transitions in twisting can be seen midway along the ookinete from the apical pole where twisting becomes less marked. G. As (F) using cryoelectron microscopy.

Towards exploring the contribution of cell shape, we first sought to determine an accurate 3D reconstruction of an ookinete. Logically, like a corkscrew, the twist of the cell will generate a similar chiral motion of the parasite through 3D space with the same handedness. However, to date, precise exploration of ookinete 3D shape has been limited to scanning electron microscopy (SEM) studies that describe an overall twisting along the longitudinal axis of these motile cells (Speer *et al*., 1974; Sinden *et al*., 1987). The twist, while clearly obvious in prior SEM work and our own images (Fig. 5A), is ambiguous with respect to handedness because of the difficulty in determining whether the system used for image capture is mirrored (see also Supplementary Discussion). Given these challenges, we used serial section Focused Ion Beam (FIB)‐EM (Bushby *et al*., 2011), a methodology that allows reconstruction of whole cells from electron micrograph sections. Reconstruction at 50 nm serial sections confirmed the twisted shape of ookinetes seen in SEM (Fig. 5B and C). With knowledge of the handedness of our imaging system and analysis (both its mirroring of the individual sections and the handedness of the Cartesian system used in assembling the 3D image) we determined that ookinete shape confirms to a left‐handed helical twist (Fig. 5C and Supplementary Movie S6).

To explore the underlying basis of this dominant left‐handed chiral shape, we attempted visualization of the intact cytoskeleton of the ookinete to determine its contribution to cell twisting. Subpellicular microtubules lie under the outer pellicle of the ookinete and are arrayed, evenly spaced, around the circumference in cross‐sectional slices through an ookinete (Fig. 5D), where they attach through to the IMC via short electron dense extensions of unknown origin (Fig. 5E, yellow arrows) (Raibaud *et al*., 2001). Previous studies with sporozoites from the related apicomplexan parasite *Eimeria acervulina* revealed a twisted or spiralling lattice of subpellicular microtubules when visualized in critical‐point‐dried whole cytoskeletal preparations (Russell and Burns, 1984). Using the same approach, visualized by negative stain and CryoEM (see *Experimental procedures*), we were able to extract the ookinete microtubule cytoskeleton and demonstrate that it too has an inherent twisted/spiral lattice made up from angled filaments (Fig. 5F and G). While chirality could not be determined (since images are by nature only two‐dimensional), the observation that the lattice spirals is consistent with an inherent linkage between intracellular architecture and a gross helical morphology.

Apicomplexan microtubules, including those of ookinetes, contain 13‐protofilaments (Raibaud *et al*., 2001; Cyrklaff *et al*., 2007), a configuration expected to give rise to straight longitudinal filaments (Amos, 2004). Their angled, spiral arrangement could conceivably arise from a skewed anchorage of each filament to the microtubule organizing centre at the ookinete apex, much like that seen in the sporozoite (Kudryashev *et al*., 2012) and/or the addition of specific protofilament‐binding proteins or linkages with the IMC (Raibaud *et al*., 2001) (Fig. 5E, yellow arrows) which would alter the direction of the overall microtubule from straight to angular (Cyrklaff *et al*., 2007). Such an organization away from longitudinal expectations would be consistent with the adaptation of the microtubule lattice for cell shape, and by extension to its role in chiral cell motion.

### Ookinete cell shape correlates with their movement in 3D space

**Figure 6:**
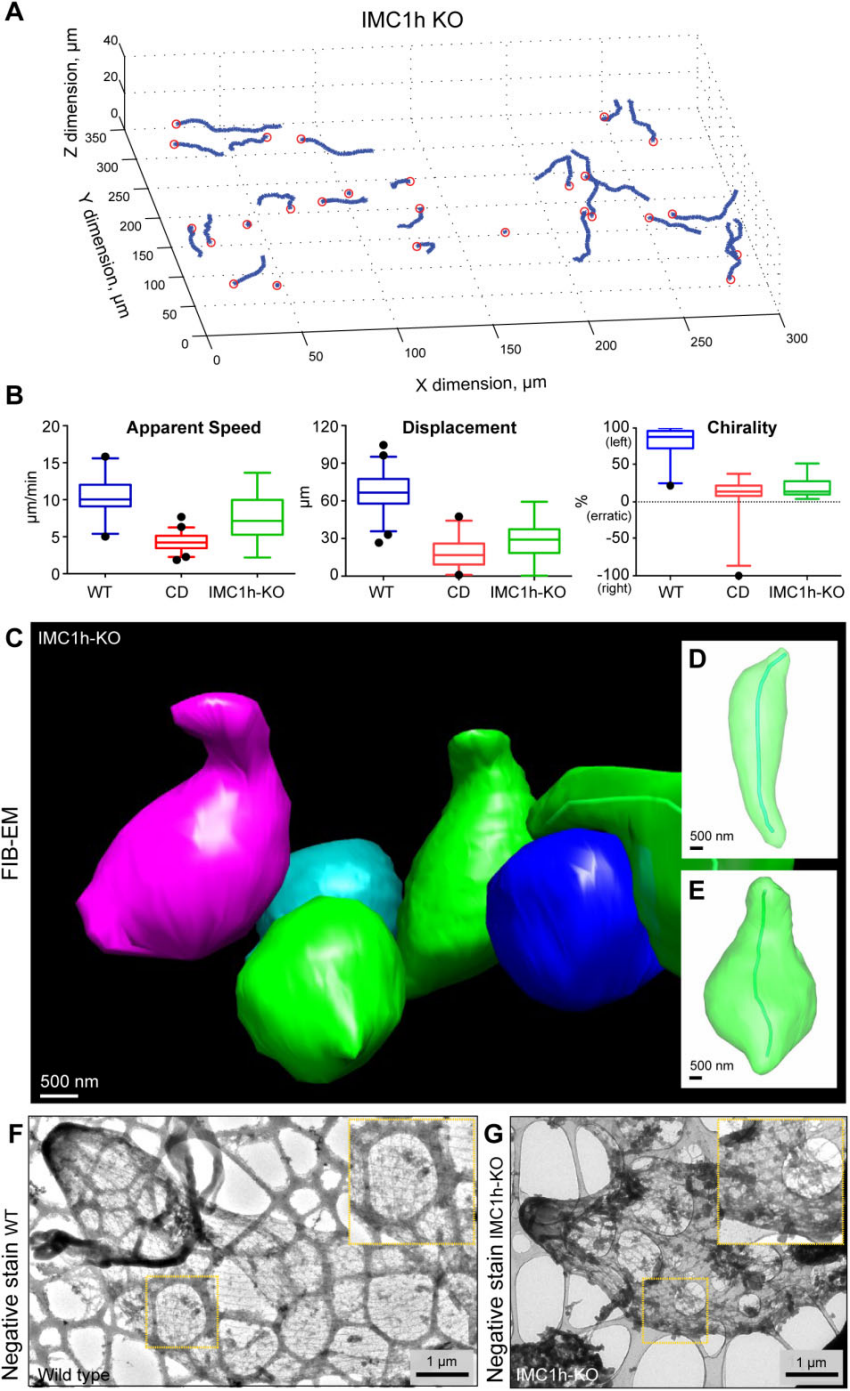
Absence of IMC1h ablates the parameters of *P**. berghei* ookinete motility. A. Reconstructed 3D tracks of IMC1h‐KO ookinetes *in vitro* demonstrate cell motility without a helical motion path. B. Mean parameters of IMC1h‐KO parasite motility in comparison to wild‐type and cytochalasin D (CD)‐treated cells. Data from three independent replicates. Boxplot error bars indicate 5th to 95th percentiles with outliers shown individually. C–E. FIB‐EM of IMC1h‐KO ookinetes reveals an absence in chiral shape. F and G. Critical point dried negative stain EM comparing wild‐type (F) and IMC1h‐KO ookinetes (G) reveals a less organized but still twisted microtubular lattice in the knockout parasites.

To address the contribution of cell shape to the parameters of ookinete movement in 3D space, disruption of parasite cytoskeleton was attempted, while leaving motor functionality unchanged. Use of microtubule‐disrupting drugs to target the microtubule cytoskeleton were precluded because of the general stability of subpellicular microtubules in Apicomplexa, their non‐dynamic nature and resistance to classical drugs such as colchicine and oryzalin (Russell and Sinden, 1981; Morrissette and Sibley, 2002) despite use at concentrations of up to 1 mM (Supplementary Fig. S1). As an alternative strategy we explored the role of proteins of the subpellicular network implicated in shaping the pellicular architecture of malaria parasites (Mann and Beckers, 2001). Recent knockout of IMC1h, an inner membrane complex protein in *P. berghei*, revealed their role in defining shape in the ookinete. Furthermore, knockout parasites gave rise to clear changes in gross cell movement (Tremp and Dessens, 2011; Volkmann *et al*., 2012). Confirming previous reports, imaging of the knockout parasite line in our 3D assay demonstrated that lack of IMC1h does not prevent movement, certainly not to the extent seen with inhibition of actin dynamics with CD. Instead IMC1h‐KO ookinetes showed a slightly reduced average apparent speed of 7.5 ± 3.1 μm min^−1^ and total displacement of 28.2 ± 16.4 μm when compared to wild‐type parasites (Table 1). IMC1h‐KO cells did not, however, follow helical motility with their chirality decreased down to effectively random (18.8 ± 11.2%). Of most interest, and unlike CD treatment, of 39 tracks analysed with the knockout none exhibited any detectable helical motion that could be used to determine properties of chiral motion (Fig. 6A and B and Table 1). Thus, changing ookinete shape markedly altered the gross parameters of motility from chiral to linear without ablating directional motility.

The shape of ookinetes following IMC disruption is visibly much rounder than that seen in wild‐type parasites (Tremp and Dessens, 2011; Volkmann *et al*., 2012) with mean length to width ratios of 2.1 ± 0.6 and 4.7 ± 4.6 respectively (Figs 5B and 6C). In support of the linkage between shape and chirality, whole‐cell FIB‐EM revealed that while an apparent curve could be detected for some parasites, no left‐handed (or any handed) twist was observable in parasite reconstructions (Fig. 6C–E, Supplementary Movies S7 and S8). Attempts at electron microscopy of critical‐point‐dried cytoskeletons were hampered by the fragility of IMC1h‐KO ookinetes to purification. Where knockout ookinetes were visible by negative stain, however, the helical lattice of the cytoskeleton was still apparent, though seemingly less organized than wild‐type (Fig. 6F and G). Given that microtubules link to the underside of the IMC (Raibaud *et al*., 2001), the breakdown in shape observed in the knockout parasite line supports the general model linking IMC and subpellicular microtubules as the core architecture framework of an apicomplexan parasites (Morrissette and Sibley, 2002), likely with IMC recruiting microtubule rather than the reverse (Morrissette and Sibley, 2002). Confounding a clear interpretation of our results with respect to how cytoskeletal organization affects motility, IMC proteins have also been linked directly to the gliding motor complex itself (Bullen *et al*., 2009). Thus, the absence of chiral motion could be derived from the gross changes in ookinete shape as well as a breakdown in motor organization. However, since directional motion is still observed (IMC1h‐KO still move along straight lines, Fig. 6A), the knockout parasite's lack of chiral motion strongly implies that cell shape, as determined by cytoskeletal architecture, is the primary driver of ookinete chiral motion.

### Why move with helical motion?

A helical trajectory results when an organism moves with constant rotational (**ω**) and translational velocity (***V***) such that these two vectors are neither parallel nor perpendicular to each other (Crenshaw, 1996). ***V*** is always tangential to the trajectory of the forward moving object, and **ω** is parallel to the axis of the helix (Fig. S3). While helical motion is very commonly seen across microorganisms, it is normally associated with flagellar beats or shape changes (Jahn and Votta, 1972; Crenshaw, 1996). For the ookinete, our analyses suggest it is instead defined by an, approximately, fixed chiral shape of the parasite. This relationship between shape and motion may relax some of the perceived constraints in gliding motor organization. For example, if force is entirely sideways/lateral (F_lat_ red arrows, Fig. S3), then parasite shape can alone still lead to progressive forward motion by the ookinete in a similar fashion to a corkscrew. Any increase in the degree of rearward force (F_rear_ blue arrows, Fig. S3), as envisaged by current thinking of the motor (Frénal *et al*., 2010), would then add to the efficiency of forward movement. Thus while the actomyosin motor may still be largely rearward directed, incorporating cell shape into the mechanics of ookinete motion may relax some of the envisaged constraints on its precise organization.

There are other benefits of helical motility, which may be key evolutionary drivers for ookinete helical motion. While we cannot exclude the possibility that helical motion is a developmental constraint (i.e. independent of motility), we can consider its influence on ookinete movement by conceptualizing a parasite well inside the midgut that can either move in a helical manner or follow a straight line along the helix axis. Given the dimensions of the midgut and the helix (midgut cross‐section width ∼ 800 μm, helix radius 2 μm), the exit points for both strategies will match closely. Seen like this, a true straight‐line strategy would appear to be beneficial: it reaches the same exit more quickly without making extra turns. With the ookinete having to battle against digestive forces of the midgut, which activate during the first 24 h (Gass and Yeates, 1979) such a delay would not be trivial. What then are the benefits of helical motion? Foremost, chiral motion is more energy efficient than linear motion through 3D space in a viscous environment (Purcell, 1977) where the chiral shape of the parasite prevents rearward slip and provides a more energy‐efficient transduction of motor force to the extracellular milieu. Helical motion is also a very good way of maintaining a straight trajectory (Jennings, 1901; Crenshaw, 1996) since the act of moving with a revolving helical axis can compensate for any external force that could influence the motile cell to deviate from a linear course (Jennings, 1901). We might therefore conclude that ookinete shape and chiral motion are beneficial for reaching the midgut epithelium both as quickly as possible and with greatest energy efficiency, while still being as close as possible to the ideal of a true straight line exit strategy.

Attempts to change viscosity parameters in the Matrigel assay here were unsuccessful (motion was ablated entirely). However, previous reports have demonstrated that ookinetes from different *Plasmodium* species move with different twisting properties (Freyvogel, 1966). Thus, the different vector‐host combinations (and associated differences in timing of maturity with respect to blood bolus viscosity, formation and density of the peritrophic matrix or properties of host epithelium) may have selected for different ookinete cell motion properties. It would be of interest to assess the pitch differences between the ookinetes from different species to test this hypothesis. Shape‐dependent left‐handed helical motion through viscous space has recently also been shown for *T. gondii* tachyzoites using a similar Matrigel matrix environment (Leung *et al*., 2014). This would suggest that the features of 3D helical motion, and its underlying cellular basis, may be a common theme across apicomplexan parasites.

While rearward force is still in action when ookinete shape is changed (Fig. 6), the potential for relaxation of constraints on motor organization clearly demands that the precise biomechanics and biophysics of force are determined for a motile ookinete, and more broadly across apicomplexan parasites in general. Specifically, studies that can directly demonstrate the precise underlying organization of the myosin motor and actin filaments are now clearly needed to enable a precise understanding of the linkage between cytoskeleton, motor force and cell shape that leads to helical motion. Ultimately, refining our understanding of how parasites move through 3D space will improve strategies for developing compounds that can hinder them, either as transmission blocking drugs or as effective treatment drugs that target other stages of the life cycle. Indeed, the precise definition of parameters of motion in the context of an *in vitro* multi‐well assay should be readily adaptable towards cellular‐based drug‐screening to explore specific inhibitors of ookinete motility or beyond to other motile apicomplexan parasite stages.

## Experimental procedures

### Parasites and mice

Four to six‐week‐old Swiss mice, housed at 22°C to 25°C on a 12 h light/dark cycle were treated with phenyldrazine (PHZ; 6 mg ml^−1^) 3 days prior to infection. Infections were carried out by intraperitonial (I.P.) inoculation using a *P. berghei* ANKA stain expressing green fluorescent protein (GFP) con (Franke‐Fayard *et al*., 2004) obtained from a donor mouse between first and fourth passages from cryopreserved stock. Parasitaemia was monitored by Giemsa smear and exflagellation quantified 3 days post infection by adding a drop of blood from a tail vein to 20 μl ookinetes medium (Moon *et al*., 2009). Percentage of gametocytaemia and number of exflagellation events were assessed in 10 viewing fields. Infected blood was collected from mice with a parasitaemia > 4% via cardiac puncture into a heparinized syringe. Ookinete cultures were produced *in vitro* by culturing in 10 ml flasks using ookinete media (Moon *et al*., 2009) and incubated at 19°C for 18 h. All experiments were conducted in compliance with local Animal Ethics Committee requirements [Baum laboratory, Animal Ethics Committees (AEC) Project 2009‐023].

### Ookinete plate‐based motility assays

GFPcon *P. berghei* ookinete cultures were allowed to settle on and invade the gel surface of 96‐well imaging plates (clear bottom, black; BD Bioscience) pre‐coated with Matrigel (BD Bioscience) diluted at a 1:1 ratio with ookinete medium (thus filtering out non‐motile fluorescent material). These were incubated at 19°C for 1 h. For drug treatments, after incubation, inhibitors made up in DMSO [100 nM Cytochalasin D (Sigma), 1 mM Oryzalin (Sigma), 1 mM Colchicine (Sigma)] were added onto the plates and parasites were incubated at 19°C for another hour. 1% DMSO was used as the negative control for all the experiments. Imaging was performed using a line‐scanning confocal LSM 5 live microscope (Zeiss) at room temperature. Fluorescence time‐lapse videos were acquired using a 20×/0.8 Plan Apochromat objective. In each video, 1 frame was taken every 2 s over a z depth of 40 μm (with 2.5 μm intervals) for the total video duration of 10 min. Laser source (488 nm) was used at transmission of 5%. The microscope was controlled by Zen v.2008 software. Videos were imported to Imaris v.7.5.1 (Bitplane) for analysis. All motility assays were recorded in triplicate wells, on at least three independent occasions (blood derived from independent mouse infections). A dosage curve for cytochalasin D treatment was determined using a Live Cell Observer microscope (Zeiss) and plate‐based Matrigel assay with average speed calculated (estimated only from net displacement) relative to DMSO control to derive an approximate ∼IC_50_ for motility inhibition.

For *in vivo* ookinete motility imaging, *A. stephensi* mosquitoes were fed with a *P. berghei* GFPcon infected blood meal. Eighteen hours post feed, mosquito midguts were dissected in phosphate buffer saline (PBS) and imaged in a poly‐d‐lysine‐coated slide chamber. Imaging settings were as above using the LSM5 Live (Zeiss).

### Cell tracking and data pre‐processing

Imaris v.7.5.1 (Bitplane) was used for automated cell localization and tracking. Spot tracking algorithm with Brownian motion model was used with the diameter of the spot set to 5 μm. A random selection of automatically reconstructed tracks was manually verified for tracking errors. Resulting 3D co‐ordinates of reconstructed tracks were exported into a Microsoft Excel file. The data were then analysed using scripts written in MATLAB v.R2012b (Supplementary Code and Data, available on request).

Tracks spanning less than 9 min (out of 10 min of total recording time) were discarded. Furthermore, only uninterrupted tracks, i.e. tracks that had no missing co‐ordinates between start and end‐points were considered for analysis. The Field Of View (FOV) spanned 40 μm in z‐direction. For analysis, tracks with all z co‐ordinates located within (2 μm; 38 μm) were considered. This was done because some ookinetes outside of the FOV along the z‐direction were incorrectly detected as being located at the FOV boundary, due to the registration of out‐of‐focus fluorescent signal.

### Parameterization of ookinete locomotion

An expanded discussion on motion path parameterization is included as Supplementary Discussion.

Apparent speed is defined as the sum of the Euclidean distances between consecutive track locations (x, y, z) divided by the duration of the track. Total displacement after 10 min is defined as the Euclidean distance between the first and the last track location. If the track spans less than 10 min (e.g. 9 min), then the distance is adjusted accordingly (e.g. multiplied by 10/9).

Chirality is the proportion of left‐handed helical primitives (*l*‐primitives), where the primitive is a sequence of four track points *P*(*i*) = (*x*, *y*, *z*) taken *d* steps apart, i.e. {*P*(*i*), *P*(*i* + *d*), *P*(*i* + 2*d*), *P*(*i* + 3*d*)}. These four points define three vectors ***A*** = *P*(*i* + *d*) – *P*(*i*), ***B*** = *P*(*i* + 2*d*) – *P*(*i* + *d*), and ***C*** = *P*(*i* + 3*d*) – *P*(*i* + 2*d*), and a cross product ***X*** = ***A*** × ***B***. If the angle *∠* (***X***, ***C***) < *π/*2, then the primitive is *l*‐primitive (Supplementary Fig. S2). For a given step *d*, a track with *N* points has *N‐*3*d* primitives, and *d‐*chirality of the track is the proportion of *l*‐primitives. Furthermore, different steps *d* = 1, … , 10 were used and maximum *d‐*chirality was noted as the track chirality.

Track period was estimated based on the Fast Fourier Transform (FFT): (i) signed differences between the x, y and z co‐ordinates of subsequent locations were computed, (ii) the FFT was computed for each sequence, and frequencies corresponding to periods larger than 1/4 of the experiment duration were discarded, (iii) the frequency component with the largest magnitude was selected, and the ratio of the maximum magnitude to the mean magnitude *R* of all components was computed for each sequence, and (iv) the frequency that had the largest ratio was selected, and the period was computed from this frequency. However, period estimation was considered failed if ratio *R* for this frequency was lower than empirically defined threshold (7 in our analysis). This could happen for tracks that do not exhibit periodicity.

In the case when period estimation succeeded, radius and pitch for a track were computed by taking three point samples from the track. A sample comprises points *P*(*i*), *P*(*i* + *T/*2), and *P*(*i* + *T*), where *T* is the estimated track period, *N* is the number of points in the track, and *i* = 1, …, *N* – *T*. Furthermore, for sample, the radius and pitch were estimated from the point co‐ordinates (Supplementary Fig. S2), and the median values of the radius and pitch were noted as the estimated radius and pitch for the track.

Ookinete tracks from *in vivo* imaging had a greater associated noise, likely arising from the background fluorescence deriving from the blood meal and immature sexual or asexual stages, making their precise parameterization more challenging. In general they were found to have longer helical periods. As such, the maximum step *d* for chirality was set to a value of 75 (*in vitro* was 10), while maximum allowed period and ratio threshold for period estimation were set to 1/2 of the experimental duration and 3 respectively, adjusted from *in vitro* observations (see above).

### Mosquito oocyst distribution imaging and analysis

*Anopheles stephensi* mosquitoes were fed on *P. berghei* infected GFPcon blood meal and sorted into pipette tips either head up or head down or left unconstrained in cages. Five days post feeding midguts were dissected into PBS and imaged for oocyst distribution with a 5× objective on a Leica widefield fluorescent microscope (Leica). Images were all orientated from anterior to posterior, oocysts positions were marked using a FIJI script and frequency distributions of oocysts along this axis were calculated.

### Electron microcopy (EM)

Ookinetes were produced *in vitro* as above and purified by nycodenz density gradient (Carter *et al*., 2003). Scanning EM and transmission EM were both as previously described (Sinden *et al*., 1987). For automated serial section using focused ion beam EM, purified ookinetes were prepared using a Glutaraldehyde/reduced osmium tetroxide/thiocarbohydrazide/OsO_4_ (ROTO) fixation method as described (Hanssen *et al*., 2013). Resin blocks were mounted on an SEM stub and placed inside the chamber of the Nova Nanolab 200 (FEI, Eindhoven, the Netherlands). Regions of interest (ROI) were located using mixed signal back scattered/secondary electrons. A 1 μm layer of platinum was deposited on top of the regions of interest and trenches cut around ROI. Data were then collected using the FEI Slice and View process with sections of 50 nm thickness. Serial sections were then re‐aligned, scaled in the Y direction to account for the data acquisition at 52° angles and segmented using the IMOD package (Kremer *et al*., 1996).

### Negative staining and CryoEM of whole pellicular complexes

Purified ookinetes were settled on a holey carbon formvar/carbon coated grid and subjected to hypotonic lysis according to a previously described method (Russell and Burns, 1984). For negative staining the ookinetes were then stained with uranyl acetate and critical point dried. For CryoEM ookinetes were plunge frozen in liquid ethane and observed under low dose condition (total electron dose of 20 e^−^/A^2^). Micrographs were recorded on a Tecnai F30 (FEI, Eindhoven, the Netherlands) at 300 kV using a Ultrascan 1000 2k × 2k camera (Gatan, Pleasanton, USA).

## Supplementary Material

**Fig. S1.** Bar graphs showing comparison between normal and drug‐treated parameters of ookinete motility in the presence of microtubule inhibitors Oryzalin (1 mM) and Colchicine (1 mM). Boxplot error bars indicate 5th to 95th percentiles with outliers shown individually.
**Fig. S2.** A schematic representation for computing ookinete motility parameters.
A. A right‐handed helical primitive (in a right‐handed Cartesian co‐ordinate system) comprise three consecutive vectors (*A*, *B*, *C*) oriented as shown in the figure. Here vectors *A* and *B* lie in the figure plane, and vector *C* is directed either away from the reader (left panel) or towards the reader (right panel). *X* is a cross‐product of vectors *A* and *B*. In the right‐handed helical primitive the angle between *X* and *C* is smaller than π/2.
B. Estimation of the radius and pitch of a single helical loop from three trajectory points *P_i_*, *P_i_*_+_*_T_*_/2_, and *P_i_*_+_*_T_*, where *T* is the estimated track period.
**Fig. S3.** The role of force in determining ookinete helical motion. A model for how organized force contributes to ookinete motion. Here ***V*** is the translational velocity and **ω** is the rotational velocity. F_lat_ and F_rear_ denote lateral and rearwards forces respectively.
**Supplementary Discussion**
**Fig. S4.** Parameter estimates are not sensitive to mild deviations in chosen threshold values. Each group represents wild‐type ookinete parameters computed using a different combination of thresholds. Boxplot error bars indicate 5th to 95th percentiles with outliers shown individually.
**Fig. S5.** The estimates of chirality for the same group of ookinetes varies with threshold, but stabilizes after the value of 10 frames. Each group represents wild‐type ookinete parameters computed using a different threshold for chirality computation. Boxplot error bars indicate 5th to 95th percentiles with outliers shown individually.
**Fig. S6.** An example that illustrates various properties of a moving ookinete (blue ellipse). The ookinete moves along some real smooth trajectory (blue curve), whereas a movie of five frames contains only five points from the trajectory (points connected by the red segments). Higher frame rates enable a better approximation of the real trajectory (bottom panel, yellow segments). In contrast, total displacement (green line) is not very sensitive to frame rate, and is characteristic of directionality of motion only.
**Fig. S7.** 3D reference object. In a right‐handed system such that the y‐axis coincides with the vertical stroke, and z‐axis is perpendicular to the plane of the symbol (and to the plane of the tissue), in the reference spatial configuration, going from point A to point B would increase both x and y co‐ordinates, while going from tissue to symbol plane would increase z co‐ordinate. After acquiring the object using a confocal microscope and inspecting the resulting z‐stack during image processing (see *Experimental procedures*), while y and z co‐ordinates increased as expected, x co‐ordinates decreased when going from A to B. This implies that in the test system the image is digitized using a left‐handed co‐ordinate system.
**Supplementary Code and Data.** Code and data is available on request including the source output from the Imaris tracking for different experiments, and the code that reads these data, which computes locomotion parameters and compares different conditions.

**Movie S1.** 40 × 3D motion path of a single *P. berghei* ookinete in Matrigel tracked using the Imaris v.7.5.1.

**Movie S2.** 20 × 3D motion of *P. berghei* ookinetes in a single well from a 96‐well plate pre‐loaded with Matrigel.

**Movie S3.** Tracking of Movie S3 using Imaris v.7.5.1.

**Movie S4.** Tracking of three dimensional motion of *P. berghei* ookinetes from an explanted *Anopheles stephensi*‐infected midgut using Imaris v.7.5.1.

**Movie S5.** Magnification of Movie S5.

**Movie S6.** Rotation of single wild‐type *P. berghei* ookinete rendered from focused ion beam (FIB) electron microscopy (EM) sections.

**Movie S7.** Rotation of single IMC1h‐KO *P. berghei* ookinete rendered from FIB‐EM sections.

**Movie S8.** Rotation of an additional IMC1h‐KO *P. berghei* ookinete rendered from FIB‐EM sections.

## References

[cmi12283-bib-0001] Amino, R., Thiberge, S., Martin, B., Celli, S., Shorte, S., Frischknecht, F., and Menard, R. (2006) Quantitative imaging of *Plasmodium* transmission from mosquito to mammal. Nat Med 12: 220–224. 16429144 10.1038/nm1350

[cmi12283-bib-0002] Amos, L.A. (2004) Microtubule structure and its stabilisation. Org Biomol Chem 2: 2153–2160. 15280946 10.1039/b403634d

[cmi12283-bib-0003] Angrisano, F., Delves, M.J., Sturm, A., Mollard, V., McFadden, G.I., Sinden, R.E., and Baum, J. (2012a) A GFP‐actin reporter line to explore microfilament dynamics across the malaria parasite lifecycle. Mol Biochem Parasitol 182: 93–96. 22138565 10.1016/j.molbiopara.2011.11.008

[cmi12283-bib-0004] Angrisano, F., Riglar, D.T., Sturm, A., Volz, J.C., Delves, M.J., Zuccala, E.S., *et al* (2012b) Spatial localisation of actin filaments across developmental stages of the malaria parasite. PLoS ONE 7: e32188. 22389687 10.1371/journal.pone.0032188 PMC3289632

[cmi12283-bib-0005] Angrisano, F., Tan, Y.H., Sturm, A., McFadden, G.I., and Baum, J. (2012c) Malaria parasite colonisation of the mosquito midgut – placing the *Plasmodium* ookinete centre stage. Int J Parasitol 42: 519–527. 22406332 10.1016/j.ijpara.2012.02.004

[cmi12283-bib-0006] Armistead, J.S., Wilson, I.B., van Kuppevelt, T.H., and Dinglasan, R.R. (2011) A role for heparan sulfate proteoglycans in *Plasmodium falciparum* sporozoite invasion of anopheline mosquito salivary glands. Biochem J 438: 475–483. 21663594 10.1042/BJ20110694 PMC3173866

[cmi12283-bib-0007] Baton, L.A., and Ranford‐Cartwright, L.C. (2005) How do malaria ookinetes cross the mosquito midgut wall? Trends Parasitol 21: 22–28. 15639737 10.1016/j.pt.2004.11.001

[cmi12283-bib-0008] Baum, J., Richard, D., Healer, J., Rug, M., Krnajski, Z., Gilberger, T.‐W., *et al* (2006) A conserved molecular motor drives cell invasion and gliding motility across malaria life cycle stages and other apicomplexan parasites. J Biol Chem 281: 5197–5208. 16321976 10.1074/jbc.M509807200

[cmi12283-bib-0009] Baum, J., Gilberger, T.‐W., Frischknecht, F., and Meissner, M. (2008) Host‐cell invasion by malaria parasites: insights from *Plasmodium* and *Toxoplasma*. Trends Parasitol 24: 557–563. 18835222 10.1016/j.pt.2008.08.006

[cmi12283-bib-0010] Berg, H.C. (1976) How spirochetes may swim. J Theor Biol 56: 269–273. 1271822 10.1016/s0022-5193(76)80074-4

[cmi12283-bib-0011] Bergman, L.W., Kaiser, K., Fujioka, H., Coppens, I., Daly, T.M., Fox, S., *et al* (2003) Myosin A tail domain interacting protein (MTIP) localizes to the inner membrane complex of *Plasmodium* sporozoites. J Cell Sci 116: 39–49. 12456714 10.1242/jcs.00194

[cmi12283-bib-0012] Bullen, H.E., Tonkin, C.J., O'Donnell, R.A., Tham, W.H., Papenfuss, A.T., Gould, S., *et al* (2009) A novel family of Apicomplexan glideosome‐associated proteins with an inner membrane‐anchoring role. J Biol Chem 284: 25353–25363. 19561073 10.1074/jbc.M109.036772 PMC2757237

[cmi12283-bib-0013] Buscaglia, C.A., Coppens, I., Hol, W.G., and Nussenzweig, V. (2003) Sites of interaction between aldolase and thrombospondin‐related anonymous protein in plasmodium. Mol Biol Cell 14: 4947–4957. 14595113 10.1091/mbc.E03-06-0355 PMC284797

[cmi12283-bib-0014] Bushby, A.J., P'ng, K.M.Y., Young, R.D., Pinali, C., Knupp, C., and Quantock, A.J. (2011) Imaging three‐dimensional tissue architectures by focused ion beam scanning electron microscopy. Nat Protoc 6: 845–858, . . 21637203 10.1038/nprot.2011.332

[cmi12283-bib-0015] Carter, V., Cable, H., Underhill, B., Williams, J., and Hurd, H. (2003) Isolation of *Plasmodium berghei* ookinetes in culture using Nycodenz density gradient columns and magnetic isolation. Malar J 2: 35. 14613512 10.1186/1475-2875-2-35 PMC293433

[cmi12283-bib-0016] Cociancich, S.O., Park, S.S., Fidock, D.A., and Shahabuddin, M. (1999) Vesicular ATPase‐overexpressing cells determine the distribution of malaria parasite oocysts on the midguts of mosquitoes. J Biol Chem 274: 12650–12655. 10212245 10.1074/jbc.274.18.12650

[cmi12283-bib-0017] Cowman, A.F., Berry, D., and Baum, J. (2012) The cellular and molecular basis for malaria parasite invasion of the human red blood cell. J Cell Biol 198: 961–971. 22986493 10.1083/jcb.201206112 PMC3444787

[cmi12283-bib-0018] Crenshaw, H.C. (1996) A new look at locomotion in microorganisms: rotating and translating. Am Zool 36: 608–618.

[cmi12283-bib-0019] Cyrklaff, M., Kudryashev, M., Leis, A., Leonard, K., Baumeister, W., Menard, R., *et al* (2007) Cryoelectron tomography reveals periodic material at the inner side of subpellicular microtubules in apicomplexan parasites. J Exp Med 204: 1281–1287. 17562819 10.1084/jem.20062405 PMC2118598

[cmi12283-bib-0020] Dessens, J.T., Beetsma, A.L., Dimopoulos, G., Wengelnik, K., Crisanti, A., Kafatos, F.C., and Sinden, R.E. (1999) CTRP is essential for mosquito infection by malaria ookinetes. EMBO J 18: 6221–6227. 10562534 10.1093/emboj/18.22.6221 PMC1171685

[cmi12283-bib-0021] Farrow, R.E., Green, J., Katsimitsoulia, Z., Taylor, W.R., Holder, A.A., and Molloy, J.E. (2011) The mechanism of erythrocyte invasion by the malarial parasite, *Plasmodium falciparum*. Semin Cell Dev Biol 22: 953–960. 22001249 10.1016/j.semcdb.2011.09.022

[cmi12283-bib-0022] Franke‐Fayard, B., Trueman, H., Ramesar, J., Mendoza, J., van der Keur, M., van der Linden, R., *et al* (2004) A *Plasmodium berghei* reference line that constitutively expresses GFP at a high level throughout the complete life cycle. Mol Biochem Parasitol 137: 23–33. 15279948 10.1016/j.molbiopara.2004.04.007

[cmi12283-bib-0023] Frénal, K., Polonais, V., Marq, J.‐B., Stratmann, R., Limenitakis, J., and Soldati‐Favre, D. (2010) Functional dissection of the apicomplexan glideosome molecular architecture. Cell Host Microbe 8: 343–357. 20951968 10.1016/j.chom.2010.09.002

[cmi12283-bib-0024] Freyvogel, T.A. (1966) Shape, movement in situ and locomotion of plasmodial ookinetes. Acta Trop 23: 201–222. 4380662

[cmi12283-bib-0025] Gaskins, E., Gilk, S., DeVore, N., Mann, T., Ward, G., and Beckers, C. (2004) Identification of the membrane receptor of a class XIV myosin in *Toxoplasma gondii*. J Cell Biol 165: 383–393. 15123738 10.1083/jcb.200311137 PMC2172186

[cmi12283-bib-0026] Gass, R.F., and Yeates, R.A. (1979) *In vitro* damage of cultured ookinetes of *Plasmodium gallinaceum* by digestive proteinases from susceptible *Aedes aegypti*. Acta Trop 38: 243–252. 43087

[cmi12283-bib-0027] Gould, S.B., Tham, W.H., Cowman, A.F., McFadden, G.I., and Waller, R.F. (2008) Alveolins, a new family of cortical proteins that define the protist infrakingdom Alveolata. Mol Biol Evol 25: 1219–1230. 18359944 10.1093/molbev/msn070

[cmi12283-bib-0028] Graf, R., Raikhel, A.S., Brown, M.R., Lea, A.O., and Briegel, H. (1986) Mosquito trypsin: immunocytochemical localization in the midgut of blood‐fed *Aedes aegypti* (L.). Cell Tissue Res 245: 19–27. 3524850 10.1007/BF00218082

[cmi12283-bib-0029] Green, J.L., Martin, S.R., Fielden, J., Ksagoni, A., Grainger, M., Yim Lim, B.Y., *et al* (2006) The MTIP‐myosin A complex in blood stage malaria parasites. J Mol Biol 355: 933–941. 16337961 10.1016/j.jmb.2005.11.027

[cmi12283-bib-0030] Gurarie, E.E., Grünbaum, D.D., and Nishizaki, M.T.M. (2011) Estimating 3D movements from 2D observations using a continuous model of helical swimming. Bull Math Biol 73: 1358–1377. 20725795 10.1007/s11538-010-9575-7

[cmi12283-bib-0031] Han, Y.S., Thompson, J., Kafatos, F.C., and Barillas‐Mury, C. (2000) Molecular interactions between *Anopheles stephensi* midgut cells and *Plasmodium berghei*: the time bomb theory of ookinete invasion of mosquitoes. EMBO J 19: 6030–6040. 11080150 10.1093/emboj/19.22.6030 PMC305834

[cmi12283-bib-0032] Hanssen, E., Dekiwadia, C., Riglar, D.T., Rug, M., Lemgruber, L., Cowman, A.F., *et al* (2013) Electron tomography of *Plasmodium falciparum* merozoites reveals core cellular events that underpin erythrocyte invasion. Cell Microbiol 15: 1457–1472. 23461734 10.1111/cmi.12132

[cmi12283-bib-0033] Heddergott, N., Kruger, T., Babu, S.B., Wei, A., Stellamanns, E., Uppaluri, S., *et al* (2012) Trypanosome motion represents an adaptation to the crowded environment of the vertebrate bloodstream. PLoS Pathog 8: e1003023. 23166495 10.1371/journal.ppat.1003023 PMC3499580

[cmi12283-bib-0034] Hegge, S., Munter, S., Steinbuchel, M., Heiss, K., Engel, U., Matuschewski, K., and Frischknecht, F. (2010) Multistep adhesion of *Plasmodium* sporozoites. FASEB J 24: 2222–2234. 20159960 10.1096/fj.09-148700

[cmi12283-bib-0035] Hellmann, J.K., Munter, S., Kudryashev, M., Schulz, S., Heiss, K., Muller, A.K., *et al* (2011) Environmental constraints guide migration of malaria parasites during transmission. PLoS Pathog 7: e1002080. 21698220 10.1371/journal.ppat.1002080 PMC3116815

[cmi12283-bib-0036] Ishino, T., Orito, Y., Chinzei, Y., and Yuda, M. (2006) A calcium‐dependent protein kinase regulates *Plasmodium* ookinete access to the midgut epithelial cell. Mol Microbiol 59: 1175–1184. 16430692 10.1111/j.1365-2958.2005.05014.x

[cmi12283-bib-0037] Jahn, T., and Votta, J. (1972) Locomotion of protozoa. Annu Rev Fluid Mech 4: 93–116.

[cmi12283-bib-0038] Janse, C.J., Mons, B., Rouwenhorst, R.J., Van der Klooster, P.F., Overdulve, J.P., and Van der Kaay, H.J. (1985) *In vitro* formation of ookinetes and functional maturity of *Plasmodium berghei* gametocytes. Parasitology 91: 19–29. 2863802 10.1017/s0031182000056481

[cmi12283-bib-0039] Jennings, H.S. (1901) On the significance of the spiral swimming of organisms. Am Nat JSTOR: 369–378.

[cmi12283-bib-0040] Jewett, T.J., and Sibley, L.D. (2003) Aldolase forms a bridge between cell surface adhesins and the actin cytoskeleton in apicomplexan parasites. Mol Cell 11: 885–894. 12718875 10.1016/s1097-2765(03)00113-8

[cmi12283-bib-0041] Jones, M.L., Kitson, E.L., and Rayner, J.C. (2006) *Plasmodium falciparum* erythrocyte invasion: a conserved myosin associated complex. Mol Biochem Parasitol 147: 74–84. 16513191 10.1016/j.molbiopara.2006.01.009

[cmi12283-bib-0042] Kappe, S., Bruderer, T., Gantt, S., Fujioka, H., Nussenzweig, V., and Menard, R. (1999) Conservation of a gliding motility and cell invasion machinery in Apicomplexan parasites. J Cell Biol 147: 937–944. 10579715 10.1083/jcb.147.5.937 PMC2169348

[cmi12283-bib-0043] Kariu, T., Ishino, T., Yano, K., Chinzei, Y., and Yuda, M. (2006) CelTOS, a novel malarial protein that mediates transmission to mosquito and vertebrate hosts. Mol Microbiol 59: 1369–1379. 16468982 10.1111/j.1365-2958.2005.05024.x

[cmi12283-bib-0044] King, C.A. (1981) Cell surface interaction of the protozoan Gregarina with concanavalin A beads – implications for models of gregarine gliding. Cell Biol Int Rep 5: 297–305. 6783325 10.1016/0309-1651(81)90228-9

[cmi12283-bib-0045] King, C.A. (1988) Cell motility of sporozoan protozoa. Parasitol Today 4: 315–319. 15463014 10.1016/0169-4758(88)90113-5

[cmi12283-bib-0046] Kremer, J.R., Mastronarde, D.N., and McIntosh, J.R. (1996) Computer visualization of three‐dimensional image data using IMOD. J Struct Biol 116: 71–76. 8742726 10.1006/jsbi.1996.0013

[cmi12283-bib-0047] Kudryashev, M., Lepper, S., Baumeister, W., Cyrklaff, M., and Frischknecht, F. (2010) Geometric constrains for detecting short actin filaments by cryogenic electron tomography. PMC Biophys 3: 6. 20214767 10.1186/1757-5036-3-6 PMC2844354

[cmi12283-bib-0048] Kudryashev, M., Munter, S., Lemgruber, L., Montagna, G., Stahlberg, H., Matuschewski, K., *et al* (2012) Structural basis for chirality and directional motility of *Plasmodium* sporozoites. Cell Microbiol 14: 1757–1768. 22776715 10.1111/j.1462-5822.2012.01836.x PMC4116596

[cmi12283-bib-0049] Leung, J.M., Rould, M.A., Konradt, C., Hunter, C.A., and Ward, G.E. (2014) Disruption of *TgPHIL1* alters specific parameters of *Toxoplasma gondii* motility measured in a quantitative, three‐dimensional live motility assay. PLoS ONE. doi:10.1371/journal.pone.0085763. 10.1371/journal.pone.0085763 PMC3906025 24489670

[cmi12283-bib-0050] Mann, T., and Beckers, C. (2001) Characterization of the subpellicular network, a filamentous membrane skeletal component in the parasite *Toxoplasma gondii*. Mol Biochem Parasitol 115: 257–268. 11420112 10.1016/s0166-6851(01)00289-4

[cmi12283-bib-0051] Mathias, D.K., Plieskatt, J.L., Armistead, J.S., Bethony, J.M., Abdul‐Majid, K.B., McMillan, A., *et al* (2012) Expression, immunogenicity, histopathology, and potency of a mosquito‐based malaria transmission‐blocking recombinant vaccine. Infect Immun 80: 1606–1614. 22311924 10.1128/IAI.06212-11 PMC3318422

[cmi12283-bib-0052] Meissner, M., Schluter, D., and Soldati, D. (2002) Role of *Toxoplasma gondii* myosin A in powering parasite gliding and host cell invasion. Science 298: 837–840. 12399593 10.1126/science.1074553

[cmi12283-bib-0053] Metzler, R., and Klafter, J. (2000) The random walk's guide to anomalous diffusion: a fractional dynamics approach. Phys Rep 339: 1–77.

[cmi12283-bib-0054] Moon, R.W., Taylor, C.J., Bex, C., Schepers, R., Goulding, D., Janse, C.J., *et al* (2009) A cyclic GMP signalling module that regulates gliding motility in a malaria parasite. PLoS Pathog 5: e1000599. 19779564 10.1371/journal.ppat.1000599 PMC2742896

[cmi12283-bib-0055] Morrissette, N.S., and Sibley, L.D. (2002) Cytoskeleton of apicomplexan parasites. Microbiol Mol Biol Rev 66: 21–38. 11875126 10.1128/MMBR.66.1.21-38.2002 PMC120781

[cmi12283-bib-0056] Munter, S., Sabass, B., Selhuber‐Unkel, C., Kudryashev, M., Hegge, S., Engel, U., *et al* (2009) *Plasmodium* sporozoite motility is modulated by the turnover of discrete adhesion sites. Cell Host Microbe 6: 551–562. 20006843 10.1016/j.chom.2009.11.007

[cmi12283-bib-0057] Murphy, G.E., Matson, E.G., Leadbetter, J.R., Berg, H.C., and Jensen, G.J. (2008) Novel ultrastructures of *Treponema primitia* and their implications for motility. Mol Microbiol 67: 1184–1195. 18248579 10.1111/j.1365-2958.2008.06120.x PMC3082362

[cmi12283-bib-0058] Perschmann, N., Hellmann, J.K., Frischknecht, F., and Spatz, J.P. (2011) Induction of malaria parasite migration by synthetically tunable microenvironments. Nano Lett 11: 4468–4474. 21910423 10.1021/nl202788r

[cmi12283-bib-0059] Pinder, J.C., Fowler, R.E., Dluzewski, A.R., Bannister, L.H., Lavin, F.M., Mitchell, G.H., *et al* (1998) Actomyosin motor in the merozoite of the malaria parasite, *Plasmodium falciparum*: implications for red cell invasion. J Cell Sci 111: 1831–1839. 9625746 10.1242/jcs.111.13.1831

[cmi12283-bib-0060] Prudencio, M., Rodriguez, A., and Mota, M.M. (2006) The silent path to thousands of merozoites: the *Plasmodium* liver stage. Nat Rev Microbiol 4: 849–856. 17041632 10.1038/nrmicro1529

[cmi12283-bib-0061] Purcell, E.M. (1977) Life at low Reynolds number. Am J Phys 45: 3–11.

[cmi12283-bib-0062] Raibaud, A., Lupetti, P., Paul, R.E., Mercati, D., Brey, P.T., Sinden, R.E., *et al* (2001) Cryofracture electron microscopy of the ookinete pellicle of *Plasmodium gallinaceum* reveals the existence of novel pores in the alveolar membranes. J Struct Biol 135: 47–57. 11562165 10.1006/jsbi.2001.4396

[cmi12283-bib-0063] Ramakrishnan, C., Dessens, J.T., Armson, R., Pinto, S.B., Talman, A.M., Blagborough, A.M., and Sinden, R.E. (2011) Vital functions of the malarial ookinete protein, CTRP, reside in the A domains. Int J Parasitol 41: 1029–1039. 21729699 10.1016/j.ijpara.2011.05.007 PMC4068204

[cmi12283-bib-0064] Russell, D.G., and Burns, R.G. (1984) The polar ring of coccidian sporozoites: a unique microtubule‐organizing centre. J Cell Sci 65: 193–207. 6715423 10.1242/jcs.65.1.193

[cmi12283-bib-0065] Russell, D.G., and Sinden, R.E. (1981) The role of the cytoskeleton in the motility of coccidian sporozoites. J Cell Sci 50: 345–359. 7033252 10.1242/jcs.50.1.345

[cmi12283-bib-0066] Sahoo, N., Beatty, W., Heuser, J., Sept, D., and Sibley, L.D. (2006) Unusual kinetic and structural properties control rapid assembly and turnover of actin in the parasite *Toxoplasma gondii*. Mol Biol Cell 17: 895–906. 16319175 10.1091/mbc.E05-06-0512 PMC1356598

[cmi12283-bib-0067] Schmitz, S., Grainger, M., Howell, S., Calder, L.J., Gaeb, M., Pinder, J.C., *et al* (2005) Malaria parasite actin filaments are very short. J Mol Biol 349: 113–125. 15876372 10.1016/j.jmb.2005.03.056

[cmi12283-bib-0068] Schüler, H., Mueller, A.‐K., and Matuschewski, K. (2005) Unusual properties of *Plasmodium falciparum* actin: new insights into microfilament dynamics of apicomplexan parasites. FEBS Lett 579: 655–660. 15670824 10.1016/j.febslet.2004.12.037

[cmi12283-bib-0069] Shahabuddin, M., Cociancich, S., and Zieler, H. (1998) The search for novel malaria transmission‐blocking targets in the mosquito midgut. Parasitol Today 14: 493–497. 17040863 10.1016/s0169-4758(98)01348-9

[cmi12283-bib-0070] Siden‐Kiamos, I., Pinder, J.C., and Louis, C. (2006) Involvement of actin and myosins in *Plasmodium berghei* ookinete motility. Mol Biochem Parasitol 150: 308–317. 17028009 10.1016/j.molbiopara.2006.09.003

[cmi12283-bib-0071] Siden‐Kiamos, I., Ganter, M., Kunze, A., Hliscs, M., Steinbuchel, M., Mendoza, J., *et al* (2011) Stage‐specific depletion of myosin A supports an essential role in motility of malarial ookinetes. Cell Microbiol 13: 1996–2006. 21899701 10.1111/j.1462-5822.2011.01686.x

[cmi12283-bib-0072] Sinden, R.E. (2009) Malaria, sexual development and transmission: retrospect and prospect. Parasitology 136: 1427–1434. 19660156 10.1017/S0031182009990667

[cmi12283-bib-0073] Sinden, R.E., and Billingsley, P.F. (2001) *Plasmodium* invasion of mosquito cells: hawk or dove? Trends Parasitol 17: 209–212. 11323288 10.1016/s1471-4922(01)01928-6

[cmi12283-bib-0074] Sinden, R.E., and Croll, N.A. (1975) Cytology and kinetics of microgametogenesis and fertilization in *Plasmodium yoelii* nigeriensis. Parasitology 70: 53–65. 1118188 10.1017/s0031182000048861

[cmi12283-bib-0075] Sinden, R.E., Winger, L., Carter, E.H., Hartley, R.H., Tirawanchai, N., Davies, C.S., *et al* (1987) Ookinete antigens of *Plasmodium berghei*: a light and electron‐microscope immunogold study of expression of the 21 kDa determinant recognized by a transmission‐blocking antibody. Proc R Soc Lond B Biol Sci 230: 443–458. 2440053 10.1098/rspb.1987.0028

[cmi12283-bib-0076] Soldati, D., Foth, B.J., and Cowman, A.F. (2004) Molecular and functional aspects of parasite invasion. Trends Parasitol 20: 567–574. 15522666 10.1016/j.pt.2004.09.009

[cmi12283-bib-0077] Speer, C.A., Rosales‐Ronquillo, M.C., and Silverman, P.H. (1974) Scanning electron microscope observations of *Plasmodium berghei* ookinetes in primary mosquito cell cultures. J Invertebr Pathol 24: 179–183. 4607047 10.1016/0022-2011(74)90008-1

[cmi12283-bib-0078] Speer, C.A., Rosales‐Ronquillo, M.C., and Silverman, P.H. (1975) *Motility of* Plasmodium berghei *ookinetes* in vitro. J Invertebr Pathol 25: 73–78. 1089734 10.1016/0022-2011(75)90286-4

[cmi12283-bib-0079] Starnes, G.L., Coincon, M., Sygusch, J., and Sibley, L.D. (2009) Aldolase is essential for energy production and bridging adhesin‐actin cytoskeletal interactions during parasite invasion of host cells. Cell Host Microbe 5: 353–364. 19380114 10.1016/j.chom.2009.03.005 PMC2683947

[cmi12283-bib-0080] Tremp, A.Z., and Dessens, J.T. (2011) Malaria IMC1 membrane skeleton proteins operate autonomously and participate in motility independently of cell shape. J Biol Chem 286: 5383–5391. 21098480 10.1074/jbc.M110.187195 PMC3037651

[cmi12283-bib-0081] Vanderberg, J.P. (1974) Studies on the motility of *Plasmodium* sporozoites. J Protozool 21: 527–537. 4138523 10.1111/j.1550-7408.1974.tb03693.x

[cmi12283-bib-0082] Vlachou, D., Zimmermann, T., Cantera, R., Janse, C.J., Waters, A.P., and Kafatos, F.C. (2004) Real‐time, *in vivo* analysis of malaria ookinete locomotion and mosquito midgut invasion. Cell Microbiol 6: 671–685. 15186403 10.1111/j.1462-5822.2004.00394.x

[cmi12283-bib-0083] Vlachou, D., Schlegelmilch, T., Runn, E., Mendes, A., and Kafatos, F.C. (2006) The developmental migration of *Plasmodium* in mosquitoes. Curr Opin Genet Dev 16: 384–391. 16793259 10.1016/j.gde.2006.06.012

[cmi12283-bib-0084] Volkmann, K., Pfander, C., Burstroem, C., Ahras, M., Goulding, D., Rayner, J.C., *et al* (2012) The alveolin IMC1h is required for normal ookinete and sporozoite motility behaviour and host colonisation in *Plasmodium berghei*. PLoS ONE 7: e41409. 22844474 10.1371/journal.pone.0041409 PMC3402405

[cmi12283-bib-0085] Wetzel, D.M., Håkansson, S., Hu, K., Roos, D., and Sibley, L.D. (2003) Actin filament polymerization regulates gliding motility by apicomplexan parasites. Mol Biol Cell 14: 396–406. 12589042 10.1091/mbc.E02-08-0458 PMC149980

[cmi12283-bib-0086] WHO (2013). World Health Report.

[cmi12283-bib-0087] Yuda, M., Sakaida, H., and Chinzei, Y. (1999) Targeted disruption of the *plasmodium berghei* CTRP gene reveals its essential role in malaria infection of the vector mosquito. J Exp Med 190: 1711–1716. 10587361 10.1084/jem.190.11.1711 PMC2195737

[cmi12283-bib-0088] Zieler, H., and Dvorak, J.A. (2000) Invasion *in vitro* of mosquito midgut cells by the malaria parasite proceeds by a conserved mechanism and results in death of the invaded midgut cells. Proc Natl Acad Sci USA 97: 11516–11521. 11027351 10.1073/pnas.97.21.11516 PMC17232

