# Supplementary material for: Quantitative analysis of Plasmodium ookinete motion in three dimensions suggests a critical role for cell shape in the biomechanics of malaria parasite gliding motility
Source: Cell Microbiol. 2014 Mar 28;16(5):734–50. doi: 10.1111/cmi.12283 (PMC4286792; doi:10.1111/cmi.12283)
Supplement: Supplementary file 1 — Fig. S1. Bar graphs showing comparison between normal and drug‐treated parameters of ookinete motility in the presence of microtubule inhibitors Oryzalin (1 mM) and Colchicine (1 mM). Boxplot error bars indicate 5th to 95th percentiles with outliers shown individually. Fig. S2. A schematic representation for computing ookinete motility parameters. A. A right‐handed helical primitive (in a right‐handed Cartesian co‐ordinate system) comprise three consecutive vectors (A, B, C) oriented as shown in the figure. Here vectors A and B lie in the figure plane, and vector C is directed either away from the reader (left panel) or towards the reader (right panel). X is a cross‐product of vectors A and B. In the right‐handed helical primitive the angle between X and C is smaller than π/2. B. Estimation of the radius and pitch of a single helical loop from three trajectory points Pi, Pi+T/2, and Pi+T, where T is the estimated track period. Fig. S3. The role of force in determining ookinete helical motion. A model for how organized force contributes to ookinete motion. Here V is the translational velocity and ω is the rotational velocity. Flat and Frear denote lateral and rearwards forces respectively. Supplementary Discussion Fig. S4. Parameter estimates are not sensitive to mild deviations in chosen threshold values. Each group represents wild‐type ookinete parameters computed using a different combination of thresholds. Boxplot error bars indicate 5th to 95th percentiles with outliers shown individually. Fig. S5. The estimates of chirality for the same group of ookinetes varies with threshold, but stabilizes after the value of 10 frames. Each group represents wild‐type ookinete parameters computed using a different threshold for chirality computation. Boxplot error bars indicate 5th to 95th percentiles with outliers shown individually. Fig. S6. An example that illustrates various properties of a moving ookinete (blue ellipse). The ookinete moves along some real smooth trajecto [file cmi-16-734-s07.pdf]

Quantitative analysis of *Plasmodium* ookinete motion in three-dimensions suggests a critical role for cell shape in the biomechanics of malaria parasite gliding motility

SUPPLEMENTARY DATA

Andrey Kan[1]*, Yan-Hong Tan[2]*, Fiona Angrisano[2,4], Eric Hanssen[5], Kelly L. Rogers[3,4], Lachlan Whitehead[3], Vanessa P. Mollard[6], Anton Cozijnsen[6], Michael J. Delves[7], Simon Crawford[6], Robert E. Sinden[7], Geoffrey I. McFadden[6], Christopher Leckie[1], James Bailey[1] and Jake Baum[2,4,7‡]

These authors contributed equally

[1] Victoria Research Laboratory, National ICT Australia (NICTA), Department of Computing and Information Systems, University of Melbourne, Victoria 3010, Australia;

Infection and Immunity Division[2] and Centre for Dynamic Imaging[3], The Walter and Eliza Hall of Institute of Medical Research, Parkville, Victoria 3052, Australia;

[4] Department of Medical Biology, University of Melbourne, Victoria 3052, Australia;

[5] Electron Microscopy Unit Bio21 Molecular Science and Biotechnology Institute and Department of Biochemistry and Molecular Biology, University of Melbourne, Parkville, Victoria 3010, Australia.

[6] School of Botany, The University of Melbourne, Parkville 3010, Victoria, Australia.

[7] Department of Life Sciences, Imperial College of Science, Technology and Medicine, London, SW7 2AZ, United Kingdom.

CORRESPONDENCE TO:

Dr. Jake Baum

‡Current address:

Department of Life Sciences

Sir Alexander Fleming Building

Imperial College London

South Kensington

London, SW7 2AZ

Email: jake.baum@imperial.ac.uk

SUPPLMENTARY FIGURES

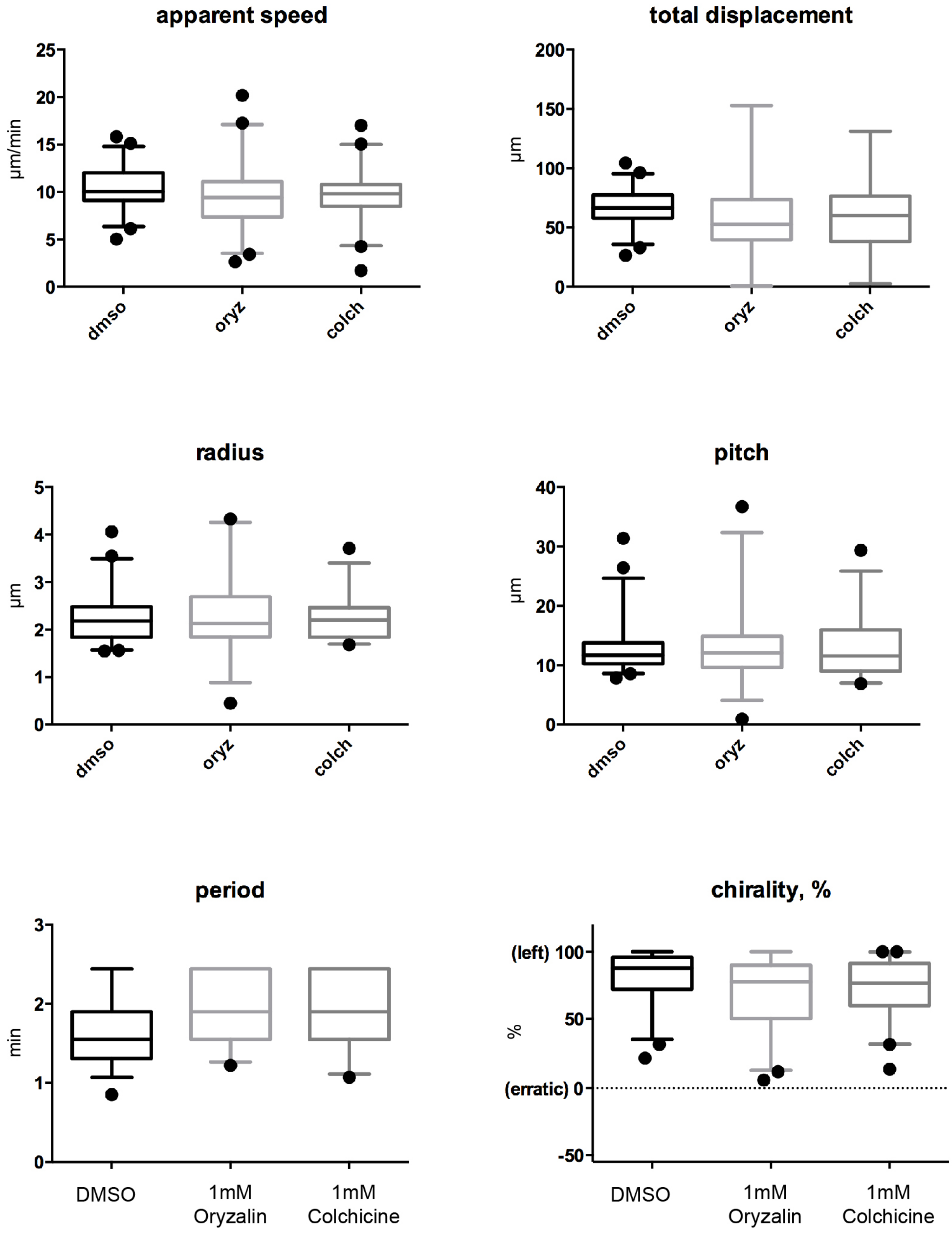

Supplementary Figure S1. Bar graphs showing comparison between normal and drug-treated parameters of ookinete motility in the presence of microtubule inhibitors Oryzalin (1mM) and Colchicine (1mM). Boxplot error bars indicate 5th to 95th percentiles with outliers shown individually.

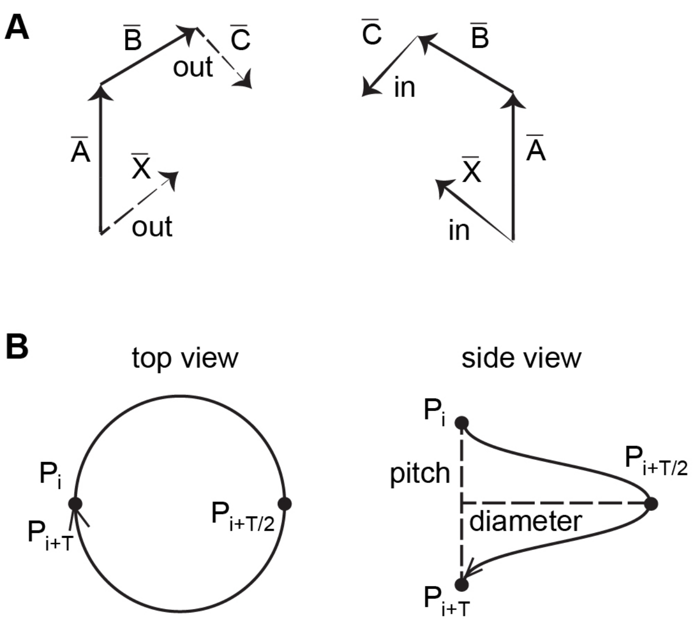

Supplementary Figure S2. A schematic representation for computing ookinete motility parameters. **A**) A right-handed helical primitive (in a right-handed Cartesian coordinate system) comprise three consecutive vectors (*A*, *B*, *C*) oriented as shown in the figure. Here vectors *A* and *B* lie in the figure plane, and vector *C* is directed either away from the reader (left panel) or towards the reader (right panel). *X* is a cross-product of vectors *A* and *B*. In the right-handed helical primitive the angle between *X* and *C* is smaller than $\pi/2$. **B**) Estimation of the radius and pitch of a single helical loop from three trajectory points P_i, $P_{i+T/2}$, and P_{i+T}, where *T* is the estimated track period.

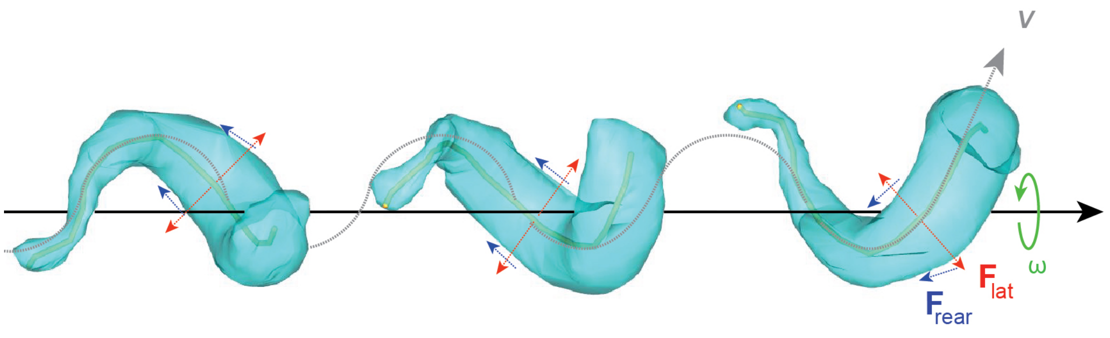

Supplementary Figure S3: The role of force in determining ookinete helical motion.

A model for how organised force contributes to ookinete motion. Here ***V*** is the translational velocity and **ω** is the rotational velocity. F_{lat} and F_{rear} denote lateral and rearwards forces respectively.

SUPPLEMENTARY DISCUSSION

Parametrization of ookinete locomotion

Parametrization here we define as the process of expressing essential properties of trajectories of a group of ookinetes (either control or treated) in a concise numerical manner, that is, using a few numbers, such as mean speed or period of motion. Qualitatively, wild type ookinetes move in a helical manner. Helical motion is a common phenomenon in biology, and at least two distinct approaches have been proposed for parametrization of the helical motion (Crenshaw, 1996, Gurarie *et al.*, 2011).

Helical trajectories can be parametrized using a curve-fitting approach (Gurarie *et al.*, 2011). This fitting is based on helix equations, but also accounts for various sources of deviations or noise from an ideal helix. The curve fitting approach is global in the sense that it attempts to fit a predefined model to the trajectory as a whole. Alternatively, a trajectory can be described using a local approach where sequences of points are extracted from the trajectory at different times. Each resulting sequence is then analyzed individually and a set of four points allows for an exact motion description for this trajectory interval (Crenshaw, 1996). The problem with the global approach is that a particular ookinete trajectory can be arbitrarily far from being a true helical track (e.g., a track can look completely erratic) in which case helical curve fitting is not justified. On the other hand, a local approach can result in unnecessary detail with each trajectory decomposed into short sequences, and each sequence having its own particular parameters.

Our approach here lies between these two. In our parametrization, apparent speed and displacement are general and not related to helical property. Chirality, on the other hand, is a continuous measure of track “helicity”. Similar to past work (Crenshaw, 1996), our calculation of chirality is closely related to Frenet trihedron, formulas that describe the kinematic properties of a particle moving along a continuous, differentiable curve in three-dimensional space. The difference here is that chirality is a single measure for a track as a whole. Finally, radius and pitch, while computed locally, explicitly rely on the assumption of a helical motion, noting that not every trajectory has an estimate of radius and pitch.

As described in Experimental Procedures, our track preprocessing and parameter estimation routines contain several constants. Some of the constants are defined by the experimental setup (e.g., the depth of the flied of view), and we justify the choice of the remaining constant values as follows. First, in the preprocessing step, we discarded tracks that span less than 0.9 of the total imaging duration. This was done so that the remaining tracks have sufficient numbers of points to infer parameters such as track period. Second, in the period estimation, if none of the discovered frequency magnitudes is considerably larger than all others, we decide that the trajectory is non-periodic. The minimum required ratio (maximum to mean magnitude) for a periodic track was empirically set to 7. We performed alternative data analyses varying these two values: the threshold for track preprocessing was set to values 0.7, 0.8, 0.9 and 1, and the period ratio threshold was set to values from 5 to 10 (i.e., around 30% deviation around the chosen value). These values gave 24 combinations, and we computed parameters of wild type ookinete locomotion using each combination of thresholds. We found that the parameter estimates are not sensitive to the choice of thresholds within the ranges tested (Supplementary Figure S4).

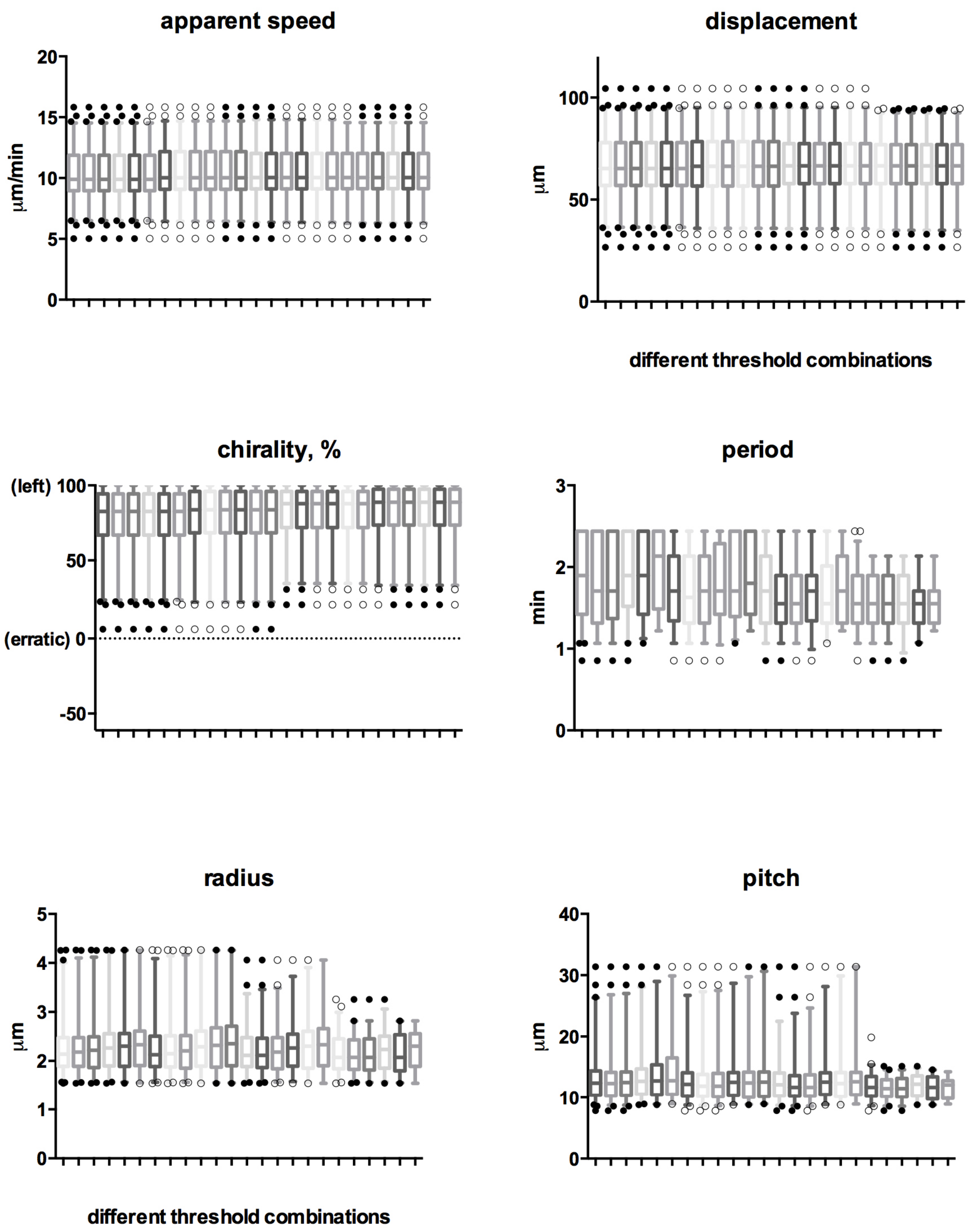

Supplementary Figure S4. Parameter estimates are not sensitive to mild deviations in chosen threshold values. Each group represents wild type ookinete parameters computed using a different combination of thresholds. Boxplot error bars indicate 5th to 95th percentiles with outliers shown individually.

For example, we compared parameter estimates between each two groups and for all parameters except for Period there was no statistically significant difference (Mann-Whitney U test, $p > 0.01$). Furthermore, for all parameters except for period, a one-way ANOVA test could not reject the

hypothesis that all combination of thresholds result in the same distribution of parameter estimates ($p > 0.1$, for the ANOVA test; we applied Fisher transformation to chirality values, and log transformation to periods, radii and pitches). For estimated parameters, some threshold combinations led to a different distribution of estimates. However, the estimated mean period was similar (between 1.5 and 2 minutes) in all groups (Supplementary Figure S4).

Finally, in the estimation of chirality, we used a threshold that determines the maximum temporal distance between subsequent points in a helical primitive. Ookinete trajectories can deviate from a true helical path due to physiology of the motion (which we attempted to measure) or noise in trajectory (e.g., measurement noise in ookinete localization). Therefore, analyzing subsequences of points taken too close in time to each other is likely to be affected by the noise. This can be addressed by using helical primitives with points taken with fewer frames apart (but still temporally close with respect to the putative helix period). We ran chirality estimation using different values (from 2 to 20 frames) of the threshold, and we found that the estimated chirality stabilizes around value of 10 (Supplementary Figure S5), and we therefore used this value in our own calculations.

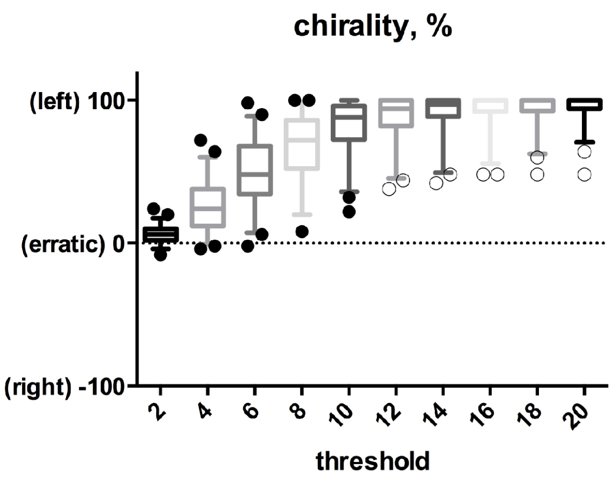

Supplementary Figure S5. The estimates of chirality for the same group of ookinetes varies with threshold, but stabilizes after the value of 10 frames. Each group represents wild type ookinete parameters computed using a different threshold for chirality computation. Boxplot error bars indicate 5th to 95th percentiles with outliers shown individually.

***Ookinetes move in random directions* in vitro**

The plot of wild type ookinete trajectories translated to a common origin suggests that ookinetes move in random directions (Figure 2C). This observation can be further quantified by looking at coordinate differences, specifically, when the difference in x, y or z coordinates for each ookinete is observed at frame 1 and frame 51 (about 1.5 minutes later). The differences (N = 52) for each of the coordinates appear to be symmetrically distributed around zero: p-values for one sample t-test for x, y and z differences are, respectively, 0.56, 0.49 and 0.28). This, in turn, indicates that there is no systematic drift in trajectories. When the same method is applied for later time points, where we looked at differences between frames 101 and 151, and finally 201 and 251, similarly, no directionality bias was found: p-values for one sample t-test for x, y and z differences (101 – 151) are, respectively, 0.54, 0.59 and 0.15; and for differences (201 – 251) are, respectively, 0.05, 0.91 and 0.44. Finally, change in directionality by comparing distributions of differences at the beginning, middle, and the end of the movies, that is, between frames (1 – 51), (101 – 151), (201 – 251) was explored. We found no difference in distributions for any of coordinates: p-values for two sample Kolmogorov-Smirnov test for x, y and z differences (1 – 51) and (101 – 151) are, respectively, 0.38, 0.89 and 0.70; and for differences (1 – 51) and (201 – 251) are, respectively, 0.17, 0.96 and 0.99. As such, analyses of trajectories by *in vitro* motile ookinetes would suggest that ookinetes move in a random direction *in vitro.* We cannot, however, exclude the possibility that in a native context external cues might bias the directionality of movement away from randomness, which we cannot test here.

Different measures of speed

Towards calculating the speed of a motile cell, it helps to consider a movie of 5 frames taken T seconds apart (Supplementary Figure S6). In reality, an ookinete recorded in the movie follows some smooth continuous trajectory (curved blue line in Figure S6) with certain real speed. Total (net) displacement of the ookinete over entirety of an assay (green straight line) is clearly not the same as the path taken by the parasite. For example, if the ookinete moves in circles, it will not go anywhere in a global sense. Whilst it is easy to estimate the total displacement: we simply

compute the difference between the positions in the first and last frames of the movie (length of the green straight line) it is much harder to estimate the real speed. This is because we do not know ookinete positions between captured frames. As such, we have to approximate the real trajectory (curved line) with straight-line segments (chain of red arrows). We then approximate real speed with what is called apparent speed (total length of all red segments divided by the total time). Seen in this light it is easy to see that higher frame rates lead to a better approximation of real speed, and to an increased apparent speed (bottom panel, yellow segments). In theory, by increasing frame rate infinitely, one gets better and better approximations and the apparent speed tends towards the real speed. In practice, if the frame rate is sufficiently high to distinguish major features of the trajectory, apparent speed gives a useful estimation of real speed. Furthermore, apparent speed comparison can be useful in studying effects on different treatments, as long as the treatment conditions were recorded at the same frame rate.

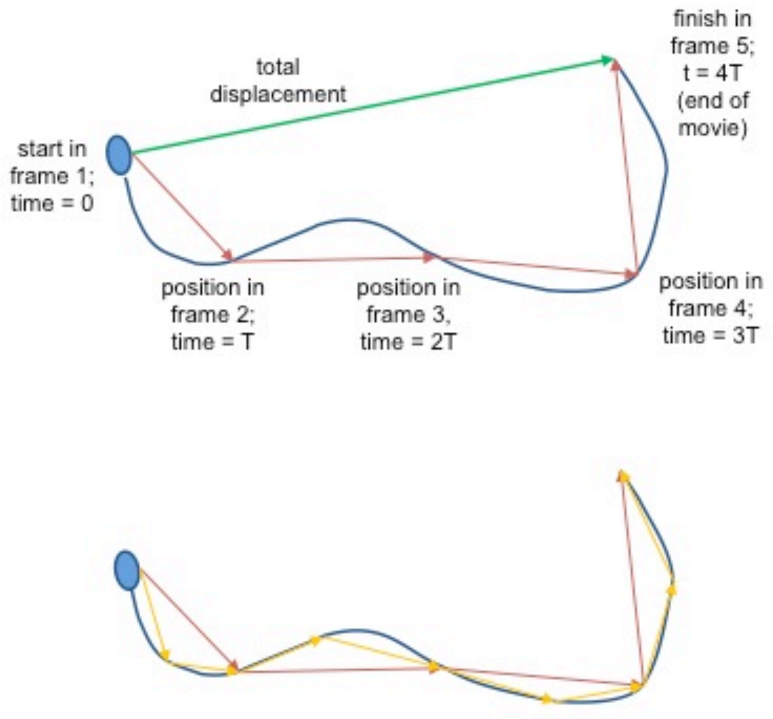

Supplementary Figure S6: An example that illustrates various properties of a moving ookinete (blue ellipse). The ookinete moves along some real smooth trajectory (blue curve), whereas a movie of 5 frames contains only 5 points from the trajectory (points connected by the red segments). Higher frame rates enable a better approximation of the real trajectory (bottom panel, yellow segments). In contrast, total displacement (green line) is not very sensitive to frame rate, and is characteristic of directionality of motion only.

Establishing the left-handedness of ookinete chiral motion

Provided track coordinates, it is possible to establish whether subsequent points locally form a right or left turn helical turn. However, the definition of handedness depends on the method of acquiring track coordinates because in 3D the coordinates can be recorded in a right- or left-handed Cartesian coordinate system. Therefore to determine the handedness of ookinete motion in our assay it was necessary to establish the relationship between the real spatial configuration of trajectories and coordinates produced after digitizing the volume with a z-stack and further processing (by respective software packages on the microscope and computer – see Experimental Procedures). I.e. it was necessary to find out whether a real right-handed track is still represented with a right-handed series of coordinates or whether it is mirrored and now represents a left-handed series of points. Of note, the optical flow of the microscope is complicated with typical microscope reference slides designed for 2D applications (e.g., to calibrate focus and scaling), but not 3D accuracy of data capture and storage.

We addressed this challenge by compiling a reference 2-slide stack (Supplementary Figure S7). The stack comprises two reference slides placed on top of each other. Viewing from the top, the first (closer, top) slide contains a symbol "1" that is inverted left-to-right. The second (bottom) slide contains a tissue section. Given this spatial configuration, and noting two points on the numerical symbol, coordinates can be assigned manually using a right hand and right-handed coordinate system. In the right-handed system, a possible coordinate assignment leads to increasing x- and y-coordinates while going from the bottom of the “1” symbol (A) to the top of the “1” symbol (B) and increasing z while going from tissue to symbol. After capturing a z-stack in our system, the z-coordinate increases from tissue to symbol, but x decreases and y increases when going from A to B. As such, the coordinates were recorded in a left-handed system, that is, the object is mirrored when represented in a right-handed coordinate system. Since chirality is computed based on the angle between a cross product of two first vectors and the third vector (see Experimental Procedures) in a right-handed system this would indicate a right-handed primitive. However, because of the mirroring, in this work, the meaning of that pattern changes to a left-handed primitive. Ookinetes therefore move with a left-handed chiral motion path.

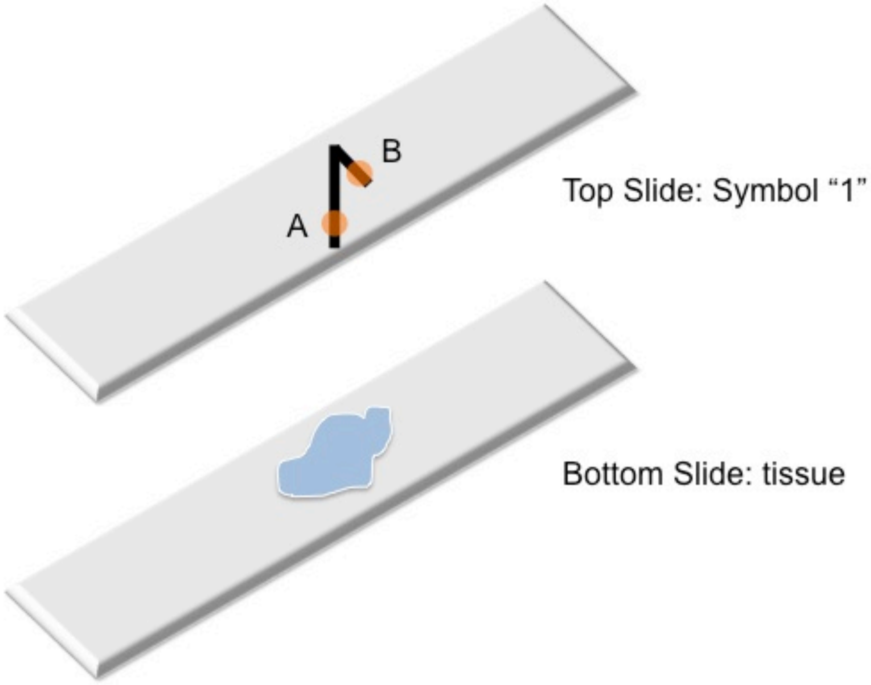

Supplementary Figure S7: 3D reference object. In a right-handed system such that the y-axis coincides with the vertical stroke, and z-axis is perpendicular to the plane of the symbol (and to the plane of the tissue), in the reference spatial configuration, going from point **A** to point **B** would increase both x and y coordinates, while going from tissue to symbol plane would increase z-coordinate. After acquiring the object using a confocal microscope and inspecting the resulting z-stack during image processing (see Experimental Procedures), whilst y- and z-coordinates increased as expected, x-coordinates decreased when going from **A** to **B**. This implies that in the test system the image is digitized using a left-handed coordinate system.

SUPPLEMENTARY MOVIES

Supplementary Movie S1

40x 3D motion path of a single *P. berghei* ookinete in Matrigel tracked using the Imaris v.7.5.1.

Supplementary Movie S2

20x 3D motion of *P. berghei* ookinetes in a single well from a 96-well plate pre-loaded with Matrigel.

Supplementary Movie S3

Tracking of Movie S3 using Imaris v.7.5.1.

Supplementary Movie S4

Tracking of three dimensional motion of *P. berghei* ookinetes from an explanted *Anopheles stephensi* infected midgut using Imaris v.7.5.1.

Supplementary Movie S5

Magnification of Movie S5.

Supplementary Movie S6

Rotation of single wild type *P. berghei* ookinete rendered from focused ion beam (FIB) electron microscopy (EM) sections.

Supplementary Movie S7

Rotation of single IMC1h-KO *P. berghei* ookinete rendered from FIB-EM sections.

Supplementary Movie S8

Rotation of an additional IMC1h-KO *P. berghei* ookinete rendered from FIB-EM sections.

Supplementary Code+Data

Code and data is available on request including the source output from the Imaris tracking for different experiments, and the code that reads this data, which computes locomotion parameters and compares different conditions.

SUPPLEMENTARY REFERENCES

Crenshaw, H.C. (1996) A New Look at Locomotion in Microorganisms: Rotating and Translating. In *American zoologist*.

Gurarie, E.E., Grünbaum, D.D. and Nishizaki, M.T.M. (2011) Estimating 3D movements from 2D observations using a continuous model of helical swimming. In *Bull Math Biol*. pp. 1358-1377.
